# Targeted delivery system for cancer cells consist of multiple ligands conjugated genetically modified CCMV capsid on doxorubicin GNPs complex

**DOI:** 10.1038/srep37096

**Published:** 2016-11-22

**Authors:** Indu Barwal, Rajiv Kumar, Suneel Kateriya, Amit Kumar Dinda, Subhash Chandra Yadav

**Affiliations:** 1 TERI University, Vasant Kunj, New Delhi, 110070, India; 2 TERI-Deakin Nano Biotechnology Centre, The Energy and Resources Institute, Darbari Seth Block, IHC Complex, Lodhi Road, New Delhi, 110003, India; 3 School of Molecular Medicine, Jawaharlal Nehru University, New Delhi, India; 4 School of Biotechnology, Jawaharlal Nehru University, New Delhi, India; 5 Department of Pathology, All India Institute of Medical Sciences, New Delhi, 110029, India; 6 Department of Anatomy, All India Institute of Medical Sciences, New Delhi, 110029, India

## Abstract

Targeted nano-delivery vehicles were developed from genetically modified *Cowpea chlorotic mottle virus* (CCMV) capsid by ligands bioconjugation for efficient drug delivery in cancer cells. RNA binding (N 1-25aa) and β-hexamer forming (N 27-41aa) domain of capsid was selectively deleted by genetic engineering to achieve the efficient *in vitro* assembly without natural cargo. Two variants of capsids were generated by truncating 41 and 26 amino acid from N terminus (NΔ41 and NΔ26) designated as F_1_ and F_2_ respectively. These capsid were optimally self-assembled in 1:2 molar ratio (F_1_:F_2_) to form a monodisperse nano-scaffold of size 28 nm along with chemically conjugated modalities for visualization (fluorescent dye), targeting (folic acid, FA) and anticancer drug (doxorubicin). The cavity of the nano-scaffold was packed with doxorubicin conjugated gold nanoparticles (10 nm) to enhance the stability, drug loading and sustained release of drug. The chimeric system was stable at pH range of 4–8. This chimeric nano-scaffold system showed highly specific receptor mediated internalization (targeting) and ~300% more cytotoxicity (with respect to FA^−^ delivery system) to folate receptor positive Michigan Cancer Foundation-7 (MCF7) cell lines. The present system may offer a programmable nano-scaffold based platform for developing chemotherapeutics for cancer.

Viral capsid proteins (CPs) were widely explored for the development of nano-medicine, drug delivery vehicles, bio-imaging/MRI contrast agents and biosensors^1^,^2^,^3^,^4^,^5^,^6^,^7^,^8^. The complete capsid of many plant viruses provides adaptability for loading of different ligands of therapeutic importance. Non-pathogenic plant viruses such as *Cowpea mosaic virus* (CPMV)^7^, *Red clover necrotic mosaic virus* (RCNMV)^9^, *Hibiscus chlorotic ring spot virus* (HCRSV)^10^, *Tobacco mosaic virus* (TMV)^11^, *Turnip yellow mosaic virus* (TYMV)^12^, *Cucumber mosaic virus* (CMV)^13^, bacteriophage MS2^14^,^15^ and Qβ^16^ were modified to carry various small molecules such as fluorescent dyes, peptides (RGD, CD 46)^5^,^17^, protein domain^18^, complex sugars, polymers^19^ and drug (doxorubicin, paclitaxel)^7^ for the development of drug delivery vehicle^5^,^10^,^12^,^15^,^20^. Various therapeutic molecules were either infused into inner cavity or bioconjugated on the capsid surface. Many of these viruses avoid the immunogenicity response in human and provide advantages in nano-medicine and drug delivery due to monodispersed size distribution and controlled assembly^3^. Among these plant viruses, CCMV capsids were much exploited for development of drug delivery vehicles due to high stability even at acidic pH and other harsh conditions^21^. The capsid is assembled from 180 protein subunits with T = 3 symmetry to form the icosahedral viral capsid^21^. The quaternary structure of CCMV shows 32 prominent capsomers arranged as pentamers (12) and hexamer (20) by carboxyl terminus interaction^22^. The N-terminus region of the CP is highly basic and binds with RNA genome of virus. These RNA binding N-terminal amino acid sequence were differentially truncated for better *in vitro* assembly without nucleic acid^23^. Though reports are available on use of this virus CP as template for manipulation by chemical conjugation and infusion of drugs/other molecules^24^,^25^,^26^,^27^,^28^,^29^, efforts are scanty on developing the modified CP based virus template into a single system for imaging, targeting, carrying and releasing drugs in cellular environment.

The present work was carried out to develop a programmable targeted chimeric nano-scaffold based drug delivery vehicle using differentially N-terminal amino acid truncated CP of CCMV with enhanced efficacy, stability and imaging properties. We report the heterologous overexpression of individual CCMV CP and their *in vitro* assembly with conjugation of various molecules such as CFSE/Alexa fluor as imaging agent, folic acid as targeting agent and doxorubicin as drug via EDC-NHS chemistry on the CP in predicted controlled proportions. The present system also describe the method for utilization of the inner cavity of the assembled nano-scaffold for packing with GNPs (10 nm) to improve the stability (stable at pH 4–8) and scope for drug conjugation like doxorubicin on GNPs to enhance the efficacy of the system yet further. The process was also optimized to control the number of molecules over the surface of the nano-scaffold system. The targeting ability and selective cytotoxicity of this vehicle were assessed using folate receptor positive (FR^+^) (MCF7) and negative (FR^−^) cell lines (HEK/HepG2).

## Results and Discussion

**Figure 1:**
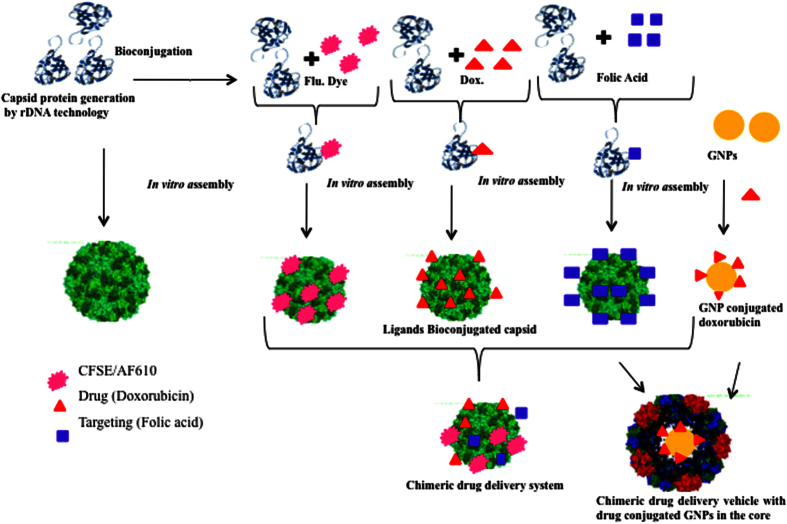
Schematic representation for the development of chimeric drug delivery system. The modified capsid proteins of CCMV virus were separately conjugated with dye (carboxyfluorescein succinimidyl ester and Alexa fluor 610), folic acid and doxorubicin. The appropriate combination of these ligand bioconjugated capsids was *in vitro* assembled keeping doxorubicin conjugated GNPs in its core to generate the nano-particulate targeted delivery vehicles.

The complete strategy to develop a chimeric multifunctional-targeted drug delivery system by *in vitro* assembly of genetically modified CCMV capsid on doxorubicin conjugated gold nanoparticles was shown in Fig. 1. This virus was selected due to its icosahedral shape, non-enveloped, size below 40 nm and without posttranslational modification. Briefly, the modified capsid proteins (CPs) were produced in *E. coli*, separately bioconjugated with folic acid, CFSE/AF610 dye and doxorubicin in functional form. These functionalized CPs in appropriate quotient were used to encapsidate doxorubicin doped GNPs in its inner cavity through *in vitro* assembly to develop the delivery vehicle. The selective *in vitro* internalization (targeting potential) and toxicity of these vehicles were validated by FR^−^ and FR^+^ HepG2/HEK and MCF7 cell lines respectively.

### Generation of complete drug delivery system

The overexpression of genetically modified capsid variants (F_1_ and F_2_) of CCMV was performed in *E. coli*. (BL21), for high yield in comparison to natural host infection^23^. The viral gene sequence of CP was codon optimized for *E. coli* using optimization software (https://eu.idtdna.com/CodonOpt) because many of viral codons were rarely used (low-usage codons) or having expression-limiting regulatory elements in *E. coli* systems^30^ (Supplementary Figure 1). The complete gene of CP was chemically synthesized because codon optimization is difficult to achieve by PCR amplification. It was computationally calculated that keeping his-tag at C-terminal of CP had minimal effect on folding and assembly^31^. Thus, these genes sequences were cloned in pET-21a vector that characteristically introduced his-tag at C-terminus.

The N-terminus amino acid sequence (25 aa) of CP were highly positive and mainly responsible for RNA interaction based assembly^23^. The deletion of these amino acid sequences completely blocked the RNA mediated assembly but had no effect on normal assembly^32^. Another 27–35 N-terminal amino acid sequences form β-hexamer responsible for providing the stability to hexameric capsomers. This enhances the *in vitro* assembly through trimer formation in suitable condition^21^. Considering this in mind and to achieve efficient virion formation in assembly condition only (to avoid assembly during overexpression and purification), two variants of N-terminal truncated capsid protein NΔ41 (F_1_) and NΔ26 (F_2_) were synthesized^23^ (Supplementary Figure 2).

**Figure 2:**
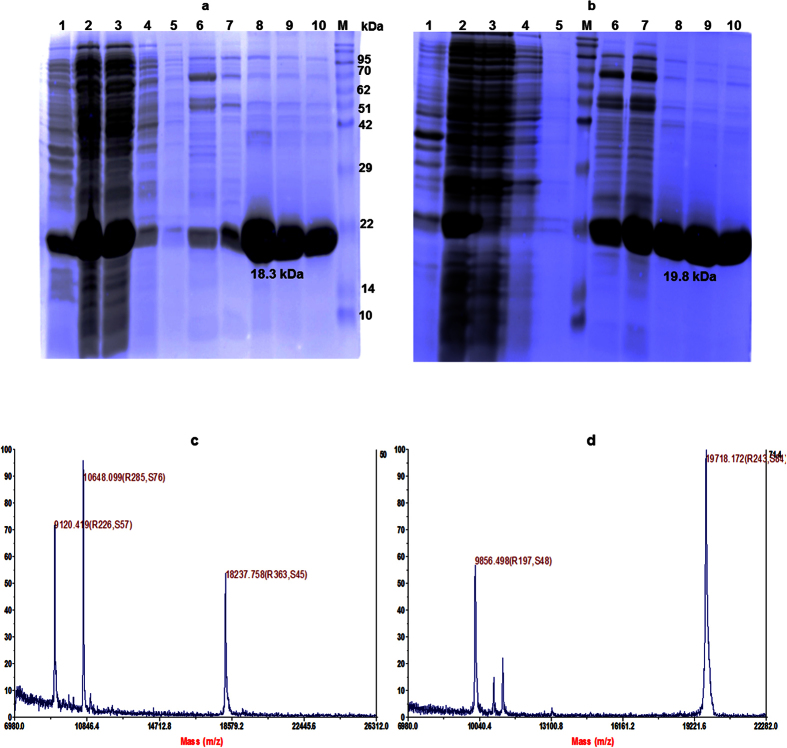
SDS-PAGE and MALDI TOF analysis of purified capsid proteins (F_1_ and F_2_). The capsid proteins F_1_ of molecular weight 18.3 kDa (**a)** and F_2_ of 19.8 kDa (**b)** were overexpressed and affinity purified using Co-NTA affinity column. Lane 1: insoluble fraction of cell lysate, lane 2: soluble fraction of cell lysate, lane 3: flow through from Co-NTA affinity column, lanes 4, and 5: PBS wash from affinity column with 1XPBS, lane 6: washed with 20 mM imidazole to remove non-specific protein bound to column; lane 7 to 10: elution fraction of 6-his tagged protein bound to Co-NTA affinity column using 250 mM imidazole, M: protein molecular weight (given in figure) standards. MALDI TOF analysis for F_1_ (**c)** and F_2_ (**d)** were given for accurate molecular mass of affinity purified samples.

**Figure 3:**
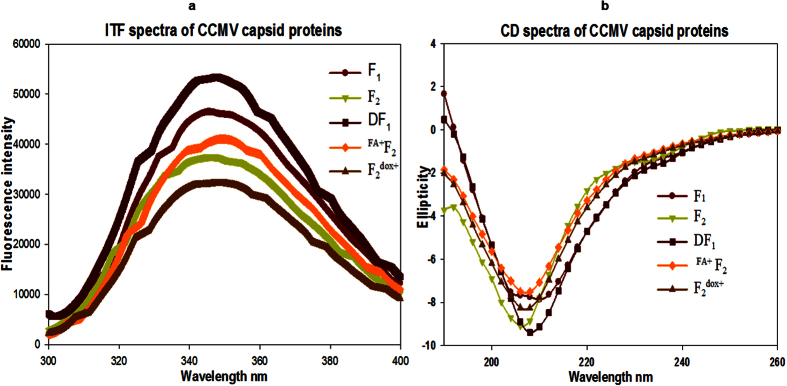
Biophysical characterization by (**a**) intrinsic tryptophan fluorescence (ITF) and (**b**) Far UV CD. For ITF measurements the 0.01 mg/ml of F_1_, F_2_, DF_1_ (CFSE/AF610 conjugated), ^FA+^F_2_ (Folic acid conjugated), and F_2_^dox+^ (doxorubicin conjugated) capsid proteins were excited at 292 nm and emission was recorded from 300 to 400 nm. The protein spectra for the capsid proteins were found near 340 nm, which confirmed the folded state of proteins. The Far UV CD spectra of F_1_ and F_2_ as well as ligands conjugated (DF_1_, ^FA+^F_2_, and F_2_^dox+^) capsid proteins (0.10 mg/ml) were taken from 190 to 260 nm at ambient temperature. The CD spectra with the negative ellipticity at 208 nm and positive peak below 200 nm suggested the folded structure of the proteins.

The N-terminal truncated CPs of CCMV were reported to be overexpressed in inclusion body using *E. coli*^23^,^33^. Many efforts had been made to overexpress these CPs in soluble fraction in our laboratory for the ease of purification, folding and scale up (Supplementary Figure 3). Based on the outcome of this finding, the overexpression conditions for both proteins (F_1_ and F_2_) were standardized using multiple permutation combination for culture medium (TB medium), induction potential (IPTG 0.3 mM) and slow growth at low temperature (16 °C). By using the entire overexpression strategies, nearly 70% of F_1_ and 100% F_2_ CPs were overexpressed in soluble fractions. SDS-PAGE and MALDI-TOF were used for molecular mass determination (Fig. 2). The yield of F_1_ and F_2_ by affinity purification was estimated as 48 mg/L and 57 mg/L culture respectively. The proteins were further identified by western blot analysis (Supplementary Figure 4). The native folding paradigm of both the proteins were prerequisite to attain the well-organized *in vitro* assembly to form delivery vehicle. This was evaluated by using intrinsic tryptophan fluorescence (ITF) and far UV circular dichroism (Far UV CD) spectroscopy^34^ (Fig. 3). ITF peak at 343 nm confirmed the native structural intactness of tryptophan, an indirect confirmation of proper folding of CP. The same spectra was shifted to 360 nm after heating (for heat induced unfolding) both the protein at 85 °C for 5 min (Supplementary Figure 5). Both the proteins showed the negative ellipticity at 208 nm and positive peak below 200 nm in Far UV CD (Fig. 3). The content of secondary structure were calculated as per Raussens method and estimated as 9.1% of α helix, 41.6% β-sheet, 12.2% turn and 37.1% random coil structure.

### *In vitro* assembly

**Figure 4:**
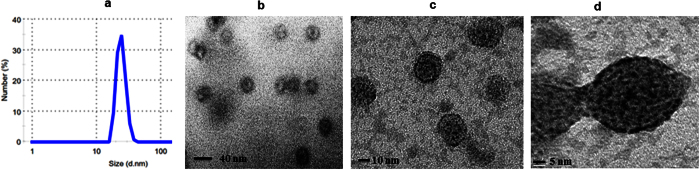
Morphological characterization of *in vitro* assembled drug delivery system. (**a)** DLS analysis of *in vitro* assembled virus nanoparticle. The capsid proteins were assembled by overnight dialysis in assembly buffer (Sodium acetate 0.1 M, sodium chloride 0.1 M, pH 4.8). (**b**,**c)** TEM micrographs of the *in vitro* assembled icosahedral virus particle at different magnifications and (**d)** higher magnification image of single *in vitro* assembled virus particle after negative staining with 0.1% PTA. Scale bar were mentioned in each figures.

The CPs were allowed to assemble individually as well as in different molar ratio (F_1_: F_2_: 1:1; 1:2; and 2:1) through dialysis against reported assembly buffer^23^. The process of monitoring the extent of *in vitro* assembly was carried out using zeta particle size analyzer and TEM imaging. The best *in vitro* assembly with uniform particle size was found in 1:2 molar ratios of F_1_:F_2_. The distribution of hydrodynamic diameter measured by DLS analysis showed monodisperse viral nanoparticles of average diameter 30.10 ± 4 nm while the TEM imaging exhibited nanoparticles of size 28 ± 4 nm (Fig. 4). The *in vitro* assembly (virion) of individual F_1_ and F_2_ resulted aggregation and poly-dispersed particle size. This was an important finding of *in vitro* assembly along with additional/modified N-terminal (cloning sequence) and C-terminal (with his-tag) amino acid sequences using N-terminal truncated capsids. This may be due to appropriate permutation of exposed N-terminal domain β-hexamer (27–35aa) of F_2_ (stabilize hexameric capsomere) and clamp sequence (44–51) of F_1_ (non-covalent dimer formation). The stability of the assembled particles (VNPs) was evaluated through TEM and particle size analyzer after incubation at various pH (from 4.0–8.0) for 24 hours. Both the study revealed the intactness of structural assembly of VNPs at pH range 4.0–8.0. At pH 4.0, 5.0 and 6.0 the capsids remain spherical with nearly same size but at pH 7.0 and 8.0, the capsid starts swelling due to pore formation (Supplementary Figure 6).

### Bioconjugation based functionalization of capsid proteins and GNPs

CCMV CPs (N-terminus truncated) was individually altered by chemical conjugation of different ligands for imaging, targeting and therapeutics on the outer surface. For maximum efficacy of targeting, imaging, drug action and release, these ligands need to be conjugated on the exposed surface of CPs. The ligands conjugation on exposed surface must have limited or no effect on the folding and assembly^35^,^36^. For this, EDC-NHS chemistry was used for the bioconjugation of ligands. This zero-length linker was employed considering its potential to protect protein from unfolding and retaining the ligand’s activity^37^. The total number and position of –COOH (6 Asp and 9 Glu) and -NH_2_ (9 Lys and 3 Arg) group containing side chains exposed on the outer surface of CCMV CPs were determined by pdb structures^21^. Among these, the most reactive/accessible side chains for chemical conjugation were assessed using two software *viz* Protscale (http://www.expasy.org/tools/protscale.html) and NetsurfP^38^. The prediction of this software revealed the most reactive amine containing side chain as Lys (42, 65, 84, 143) and Arg (83). Similarly, Asp (128, 132, 153) and Glu (35, 63, 166, 174 and 176) were predicted for carboxylic group containing side chain. The Protscale tool computes the amino acid profile taking the consideration of different parameters such as polarity, accessibility, hydrophobicity, and percentage buried residues by scanning the protein sequence and structure. Similarly, NetsurfP works with two neural network ensembles. First network predict the sequence and secondary structure while second network predict the reactive surface exposure on selected amino acids residue. The controlled conjugation of ligands on CP through these group(s) play an important role to reduce the chances of steric hindrance during *in vitro* assembly to form a protein based drug delivery vehicle.

### Bioconjugation of fluorescent dyes (CFSE and AF610) on capsid proteins

The exposed and reactive side chains of Lys residue of CPs were labeled with either carboxyfluorescein succinimidyl ester (CFSE) or Alexa fluor 610 NHS ester (AF610) for fluorescent bio-imaging. Both the fluorescent molecules were selected and used for different experimental imaging to avoid the cross fluorescence. Activated fluorescent dyes (for bioconjugation) in the form of succinimidyl ester were used for covalent conjugation with CPs. The suitable incubation time and bioconjugation efficiency was determined by using work curve scan plot for absorbance and fluorescence as well as HPLC quantification. This work curve scan plot was simultaneous scan of absorbance (200–800 nm) and fluorescence with known concentration of ligands and CPs. The bioconjugation was further confirmed by fluorescence (ex at 492 nm and em at 520 nm) in CFSE dye conjugated and extensively dialyzed CP. Similarly a peak at 630 nm was observed in AF610 conjugation after excitation at 610 nm. Analogous fluorescence was not observed in non-conjugated CP. The number of dye per capsid was calculated by taking the absorbance and fluorescence scan based standard work curve scan spectra. The absorbance/fluorescence of known concentrations of dye were taken and the absorbance/fluorescence of dye conjugated protein was measured. Number of dye per protein molecule was calculated based on the molar concentration of CP. It was estimated that about 3 (CFSE) and 5 (Alexa fluor) molecules were individually conjugated with CP (F_1_) by both methods. CP and dyes bioconjugation were also evaluated using SDS-PAGE at different incubation time. Only dye conjugated protein produces fluorescence in SDS-PAGE. The fluorescent band on the SDS-PAGE provides direct evidence of bio-conjugation of dyes on CP. The non-conjugated CP showed the single band in the same SDS-PAGE after CBB staining along with dye conjugated proteins. The folding of CP after dye bioconjugation is critical for *in vitro* assembly. ITF and far UV CD spectra were taken to confirm the folding pattern. An equal ellipticity negative peak (at 208 nm) and ITF (at 341 nm) of dye conjugated CPs confirmed the intact folded structure (Fig. 3). DLS and TEM imaging study confirmed the intactness of *in vitro* assembly of dye conjugated protein along with other assembling condition (Supplementary Figure 7).

### Bioconjugation of folic acid (FA) on capsid proteins

To achieve the targeting capability for subcutaneous cancer, folic acid (FA) was selected due to differential overexpression of folate receptor (FR) on the surface of these cells^10^,^20^. FA functionalization of viral nanoparticles was reported to enhance cellular uptake by specific tumor through receptor-mediated endocytosis^39^. Folic acid was conjugated on F_2_ by activation of γ-carboxylic group of folic acid by EDC-NHS chemistry^10^. The FA interacted through pterin group for anchoring folate in the binding pocket of FR while the glutamate group is available for bioconjugation reaction as reported earlier^40^. Bioconjugation of folic acid with CPs were confirmed by UV absorption spectra based work curve model and HPLC quantification similar to the dye bioconjugation. The absorption scans of non-conjugated protein showed a single absorption peak at 280 nm, while FA had two peaks at 285 nm and a characteristic peak at 363 nm. FA conjugated CP after extensive dialysis showed the characteristic peak of FA at 363 nm. The presence of FA characteristic peaks at 363 nm in FA conjugated CPs (^FA+^F_2_) confirmed the conjugation of FA on the CPs. This peak was not observed on incubation of FA with CPs without EDC/NHS reaction after the extensive dialysis. The average numbers of FA molecule bioconjugated on CP were quantified similar to dye conjugation using absorption scan based work curve method at 363 nm. The equivalent absorbance intensity peak of ^FA+^F_2_ at 363 nm were compared with FA calibration curve and known concentration of CP to quantify the average number of FA per CP. The average two molecules of FA per CP (bioconjugation efficiency) were conjugated using this approach. ^FA+^F_2_ protein showed intact band providing the integrity of the CP after conjugation. The folding was confirmed by using CD and ITF. The CD peaks at 210 nm and ITF at 342 confirms the native folded structure of both CPs (Fig. 3). FA conjugated CPs were also assembled to form the viral nanoparticles like structure under similar assembly condition. This was confirmed by DLS and TEM analysis (Supplementary Figure 8).

### Bioconjugation of doxorubicin on capsid proteins

Doxorubicin is well known anticancer drug which interacts with nucleic acid to block the transcription/translation by inhibiting the progression of topoisomerase II^41^. The accessible carboxylic group of acidic amino acids (Glu and Asp) of F_2_ protein was activated by EDC/NHS for the bioconjugation of doxorubicin through C-3′ amino terminus of daunosamine sugar. This group was found to be most active and stated towards the distal end of DNA binding site that facilitates DNA intercalating properties of doxorubicin after bioconjugation. This was also confirmed by various reports about the persistency of functional activity of doxorubicin after bioconjugation with viral capsid protein^7^,^35^. The absorption spectra based work curve model confirmed the chemical bioconjugation displaying the doxorubicin specific characteristic peaks at 480 nm in F_2_. This peak was absent in non-conjugated F_2_ as well as co-incubated with CP without EDC/NHS reaction after rigorous dialysis. We have standardized a method to control the number of dox per CP by just adjusting the molar ratio of doxorubicin and CP. However, for the current report we used average four doxorubicin/CP (F_2_^dox+^), which was also quantified by HPLC analysis. Bioconjugation was also confirmed by fluorescence of doxorubicin conjugated protein in unstained SDS-PAGE and 1% agarose gel. The conjugated protein showed the fluorescence at the same position of CPs. The structure function relation of F_2_^dox+^ was further confirmed by the far UV CD and ITF measurement. ITF showed the peak at 340 nm and CD peak at −208 nm of F_2_^dox+^ (same as F_2_). This provides evidence for folded structure of F_2_^dox+^ (Fig. 3). TEM and DLS analysis revealed that *in vitro* assembly of F_2_^dox+^ remain unchanged in standard assembling conditions.

### Functionalization of GNPs by doxorubicin

**Figure 5:**
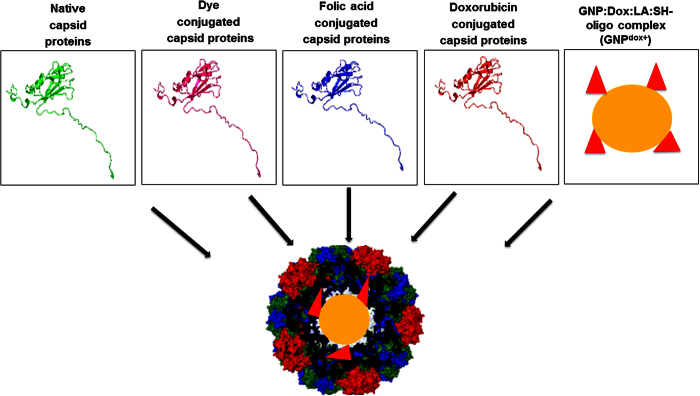
Schematic representation of synthesis of GNP^dox+^:^FA+^DDS^dox+^. The native, dye, folic acid, and doxorubicin conjugated capsid proteins in appropriate (defined) concentration were *in vitro* assembled on GNP^dox+^ to generate the chimeric and targeted delivery vehicle.

We have tried the templated assembly of functionalized CP around the doxorubicin conjugated gold nanoparticles of size10 nm as a non-natural cargo (Fig. 5). This size was appropriate to fit inside the core cavity (18 nm) of *in vitro* assembled delivery vehicle of size 28–32 nm 21 (www.viperdb.scripps.edu). For efficient encapsidation, the GNPs were functionalized with RNA probe to mimic as natural cargos to enhances the nucleation as origin of assembly (OAS)^42^. Contrary to the previous report, we have randomly selected many 15–30 mer oligo probes which showed similar templated encapsidation^42^. The most suitable 18mer 5′ end thiolated probe (CCCGACCTAGCCGACGAC) in 1:200 (GNPs:Oligo) was used based on theoretical calculations of surface area of GNPs (10 nm). The negatively charged oligo helps in interaction with the positively charged lysine side chain of CP in absence of RNA binding domains.

Further to improve drug-loading efficiency, GNPs were co-conjugated with doxorubicin drug using lipoic acid (LA) as linker. The lipoic acid was first conjugated (through Sulphur containing ring) with GNPs and unbound LA was removed by dialysis. The carboxylic group of LA (on another end) was explored to bioconjugate doxorubicin by EDC-NHS chemistry to form GNP-LA-dox complex, denoted as GNP^dox+^ in this report. The morphological characterization of GNP^dox+^ revealed the monodisperse nanoparticles (10 nm) by TEM and zeta size analysis having zeta potential −24 mV. The zeta potential after RNA conjugation was supposed to increase but this effect was nullified by the conjugation of positively charged doxorubicin molecule. The bioconjugation efficiency of doxorubicin was found to be 25% as calculated by using absorbance and fluorescence (Supplementary Figure 10).

### Synthesis of chimeric delivery vehicle GNP^dox+^:^FA+^DDS^dox+^ by *in vitro* assembly

**Figure 6:**
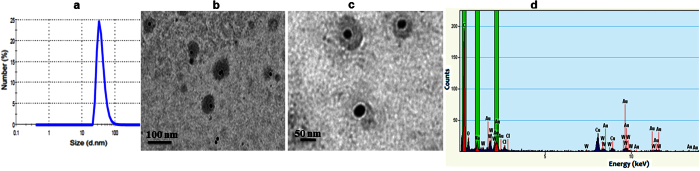
Morphological characterization of GNP^dox+^:^FA+^DDS^dox+^. (**a)** DLS measurement (**b)** and (**c)** TEM image and (**d)** EDX analysis of synthesized GNPs encapsidated complete chimeric drug delivery system (GNP^dox+^:^FA+^DDS^dox+^). The TEM image was taken without negative staining to visualize the GNPs encapsidation with protein capsid (less dark surrounding zone) as well as with staining. The EDAX analysis showed the peaks for gold which confirms the GNPs in the solutions.

Complete drug delivery vehicles were assembled with all the functionality containing CP under assembling conditions (Fig. 5). The final 1:2 molar ratio of F_1_:F_2_ including native (F_1_ and F_2_), dye (F_1_), folic acid (^FA+^F_2_) and doxorubicin (F_2_^dox+^) conjugated CPs were *in vitro* assembled in presence of GNP^dox+^ for the effective synthesis of chimeric delivery vehicles (Table 1). The oligo conjugated GNP^dox+^ act as non-natural cargo as well as additional source for drug. This assembled DDS serve as delivery vehicle with targeting, bio-imaging and drug with sustainable release. The oligo molecules on GNPs provide more stability to the whole system by ionic interaction. The GNP^dox+^ were completely wrapped by CPs, thus endow the nanoparticles biocompatibility and shield from external environment. All the possible combinations of native and ligand bioconjugated F_1_ and F_2_ CP were used to find the best combination for *in vitro* assembly with respect to size, shape, stability and monodispersity of the synthesized DDS (Supplementary Figure 11a). The desired amounts of the CPs (native and ligand bioconjugated) with and without GNP^dox+^ were dialyzed against the assembly buffer to synthesize these variants. The final concentration of doxorubicin bio-conjugated on CP and GNPs were ~60 ± 2 μM and ~80 ± 4 μM respectively. The morphological characterization reveals the formation of these variants in each case after overnight dialysis (Fig. 6). The GNPs encapsidated capsid showed better stability in storage condition (4 °C) up to four months as evidence absorbance scan (Supplementary Figure 11b).

### Release of doxorubicin from different DDS systems

**Figure 7:**
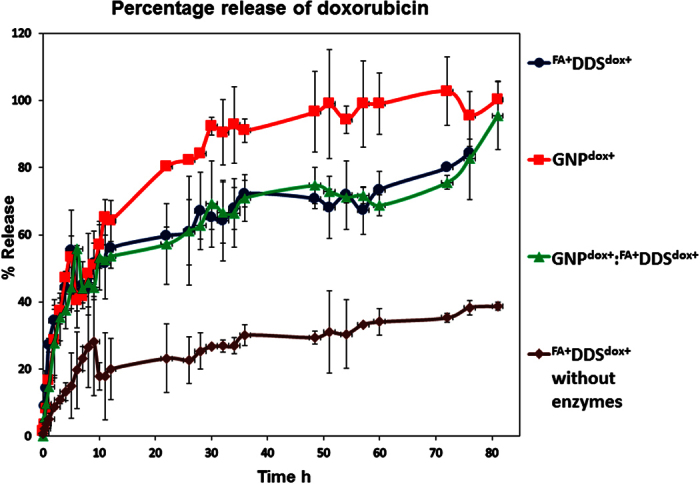
*In vitro* release of doxorubicin from different formulation: The release profile of GNP^dox+^ showed 50% release in 9 hrs and then gradual release of doxorubicin upto 4 days. The DDS^dox+^ and GNP^dox+^:^FA+^DDS^dox+^ showed similar release profile in presence of enzymes while the release study from DDS^dox+^ without enzyme showed very less 38% release of doxorubicin.

The release of doxorubicin was separately studied from GNP^dox+^, chimeric delivery vehicle (GNP^dox+^:^FA+^DDS^dox+^) and without GNP^dox+^ encapsidation (DDS^dox+^). The *in vitro* release study was separately performed in a simulated lysosomal condition in presence of lysosomal protease at acidic pH (4.8) and in PBS buffer. The lysosomal proteases were used to mimic the actual proteolytic digestion of CP to release doxorubicin after cellular uptake. These delivery vehicles were first accumulated in lysosomes inside the cells after receptor mediated (folic acid) endocytosis. The proteins of this nanomedicine were gradually degraded by proteolysis to release covalently attached doxorubicin from the capsid and GNP^dox+^. Due to this prolonged sustained release, covalent conjugation of doxorubicin offers an advantage over free drug infusion based drug delivery vehicles reported for the RCNMV, CMV based drug delivery vehicles^9^,^13^. By this way, low dose of drug may show better therapeutic attributes. The release of doxorubicin was studied by keeping individual GNP^dox+^, GNP^dox+:^ ^FA+^DDS^dox+^, and ^FA+^DDS^dox+^ along with lysosomal extract in a dialysis bag. The released doxorubicin was relatively quantified by fluorescence (excitation at 480 nm, and emission at 592 nm) using predefined standard work curve model. This study confirmed that doxorubicin was released in a sustained manner at physiological lysosomal pH (4.8). The half release of drug was observed in 9.0 h while it took 4 days for completely release (Fig. 7). All these complex (GNPs^dox+^, GNPs^dox+^: ^FA+^DDS^dox+^, and ^FA+^DDS^dox+^) did not show any significant initial burst release, which was the major problem with infusion based viral drug delivery vehicles. The *in vitro* release study without the use of proteolytic enzymes at pH 7.4 reveals no such significant release of doxorubicin. This confirms that delivery vehicle may release insignificant amount of doxorubicin in blood circulation after intravenous injection. This result has a lot of implication for *in vivo* study, as there were no losses of drug in blood circulation. Due to sustained and complete release of drug, this chimeric drug delivery system may provide an attractive nano-platform for drug delivery.

### Targeted *in vitro* internalization assay

**Figure 8:**
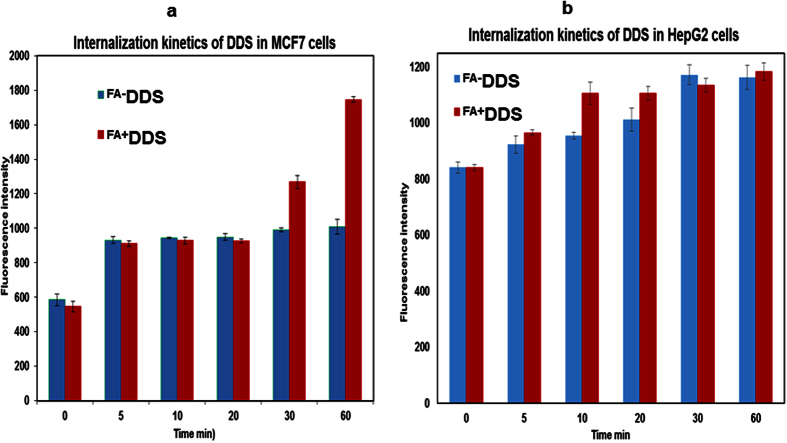
Quantitative estimation of internalized drug delivery system in (**a**) MCF7 cells (Folate receptor positive FR^+^) and (**b**) HepG2 cells (Folate receptor negative FR^−^). (**a**) Without doxorubicin-conjugated folic acid positive (^FA+^DDS-red in graph) and negative (^FA−^DDS-blue in graph) drug delivery systems were used for study. Increased ^FA+^DDS intake with time in MCF7 cells implicating the intake through receptor mediated endocytosis while cells incubated with ^FA−^DDS (blue) did not show increase in internalization due to lack of folic acid on surface of DDS up to 60 min. (**b**) There was insignificant change in fluorescence of HepG2 cells along with both ^FA+^DDS (red) and ^FA−^DDS (blue) with time. This was due to negligible folic acid receptor on the surface of HepG2 cell lines. The initial static fluorescence were due to adsorption on the cell surface in both the cell lines.

**Figure 9:**
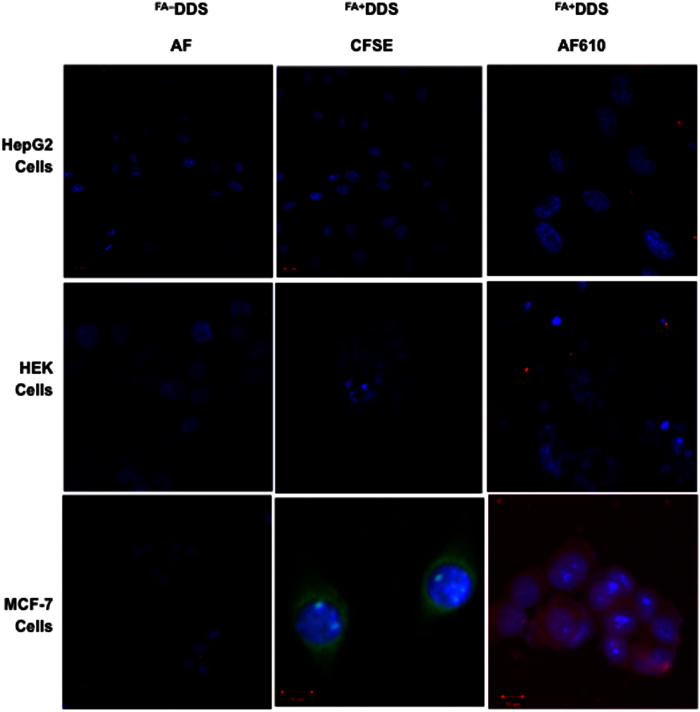
*In vitro* cellular uptake of ^FA−^DDS and ^FA+^DDS (CFSE dye conjugated) and ^FA+^DDS (AF610 dye conjugated) by FR^−^ (HepG2, HEK) and FR^+^ (MCF7) cells: Confocal images demonstrating internalization of ^FA−^DDS and folate targeted ^FA+^DDS in HepG2, HEK and MCF7 cells after 60 min of incubation at 37 °C in all experiments. The micrographs confirmed ^FA−^DDS as well as ^FA+^DDS were not delivered to FR^−^ HepG2 and HEK cells as there was no observable fluorescence while there was selective intake of ^FA+^DDS in FR^+^ MCF7 cells as compared to ^FA−^DDS depicted by high fluorescence intensity. Cells were labeled with a blue fluorescent nuclear stain (Hoechst 3342) while the DDS were labeled with AF610 (red fluorescence) or CFSE (green fluorescence). Scale bar = 10 μm.

*In vitro* cell line internalization study was performed in folate receptor positive (FR^+^) MCF7 and FR^−^ HepG2/HEK cell lines to determine the efficacy of developed drug delivery system (^FA+^DDS^dox−^)^43^. FR^+^ cell line has potential for selectively uptake of folic acid containing nano-medicine by receptor-mediated endocytosis^13^,^20^. This uptake was experimentally quantified by measuring fluorescence of CFSE/AF610 dye conjugated with nanomedicine in cellular extract. Both selected dyes (CFSE and AF610) possess unique spectral characteristics, which was easily determined qualitatively (confocal microscopy) and quantitatively (fluorescence measurement). The internalization study of fluorescently labeled ^FA+^DDS^dox−^ and ^FA−^DDS^dox−^ (4.2 μM) on MCF7 showed 1.7 and 2.5 times more fluorescence intensity after 30 and 60 min incubation respectively (Fig. 8). Confocal laser scanning microscopy imaging at various time intervals provided supporting evidence for folic acid mediated targeted uptake of ^FA+^DDS^dox−^ by MCF7 cells. The significant increases in confocal fluorescence with time (0–1.0 hour) were only found in MCF7 incubated with ^FA+^DDS^dox−^ while remain identical with ^FA−^DDS^dox−^ (Fig. 9). This indicated that ^FA+^DDS^dox−^ can efficiently deliver drug to targeted FR^+^ cells through receptor-mediated endocytosis. The uptake of both types of DDS between 0–20 min of incubation was similar in fluorimetry quantification and confocal imaging. The internalization of these vehicles on FR^−^ HepG2/HEK cells were not increased with incubation times up to 60 minutes (Fig. 8). The fluorescence was not detected in confocal imaging in these cell lines (Supplementary Figure 12). This confirm that uptake of ^FA−^DDS^dox−^ was not mediated through receptor mediated endocytosis. The doxorubicin was not conjugated in these vehicles to avoid the drug induced cellular toxicity for quantitative determination of internalization.

Targeted drug-delivery strategies have made an intense impact in biomedical field to deliver drugs in the finest dosage for extended periods to increase the efficacy, enhancing patient compliance and possibility to use highly toxic, inadequately soluble or comparatively unstable drugs. This vehicle was engineered to preferably target cells and therefore minimize drug loss and toxicity associated with delivery to undesired tissues. For effective intravenous delivery, the interaction between DDS with other cells, such as macrophages must be controlled to avoid the immunogenic response. These plant virus based delivery vehicles can be easily modified by changing surface properties. To remove non-specific protein adhesion and decrease uptake by macrophages, these vehicles may be easily functionalized using poly(ethyl- ene glycol) (PEG) and polysaccharides like protein repellent materials^44^. These non-adhesive surface coatings increase the circulation time of the nanoparticles and reduce toxic effects associated with foreign protein based immunological response^3^,^45^.

### Competitive inhibition assay

**Figure 10:**
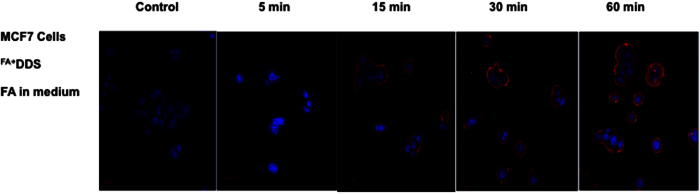
Competition inhibition assay: Confocal micrographs of competition assay showing inhibition of cellular uptake in folate receptor blocked MCF7 cells. The ^FA+^DDS was not taken up by the MCF7 cells and observed only on the cell surface due to blockage of folate receptor by free FA in the medium.

**Figure 11:**
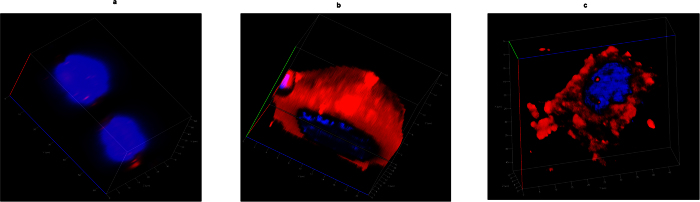
Confocal Z-scan of (**a**) ^FA−^DDS (**b**) ^FA+^DDS with normal medium and (**c**) ^FA+^DDS having excess of folic acid in the medium (competition inhibition assay) to competitively block the folate receptor on MCF7 cells. These DDS were incubated for 60 minutes in the selected medium. The ^FA−^DDS showed negligible fluorescence after rigorous washing. However, ^FA+^DDS with normal medium (no excess FA in medium) showed homogenous fluorescence distribution while the fluorescence was recorded only on the surface of cells containing excess of FA in the medium.

Competition assay was performed to further validate the receptor-mediated uptake of ^FA+^DDS^dox−^ into FR^+^ MCF7 cells. Excess amount of folic acid was added in the medium to competitively bind with FA receptors on cell surface. The binding efficiency of free FA was reported to be higher than drug delivery system conjugated folic acid^20^. This resulted in the reduction of internalization of ^FA+^DDS^dox−^ in MCF7 cells as compared to control MCF7 cells (Fig. 10). Excess free folic acids in the medium reduce the internalization of ^FA+^DDS^dox−^ for cellular uptake. This implicated that uptake of DDS was folic acid mediated in MCF7 cells. The Z-scan confocal micrograph showed uniform distribution of ^FA+^DDS^dox−^ in the control cells while delivery vehicles were entrapped on the cell surface in excess FA containing medium (Fig. 11). The reduction in internalization by this competitive binding of FA than ^FA+^DDS^dox−^ in MCF7 exhibited a layer on the cell surface similar to that of FR^−^ HEK/HepG2 cells.

### Therapeutic efficacy by XTT Assay

**Figure 12:**
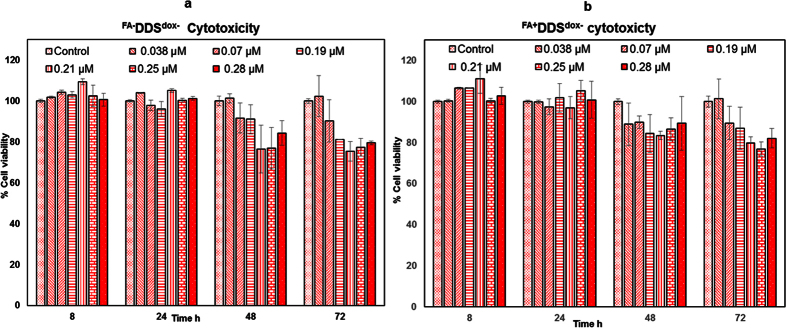
Cytotoxicity of DDS to MCF7 cells by XTT Assay. The assay was performed using selected concentration (given along with the figure) of (**a)** ^FA−^DDS^dox−^ and (**b**) ^FA+^DDS^dox−^. The comparative cytotoxicity effect of with (internalized) and without (not internalized) folic acid on DDS was evaluated by using the concentrations range of 0.038 to 0.28 μM (DDS) with selected time (from 8.0 hour to 72.0 hours). The cell viability was reported taking untreated control cells as 100%. Results were presented as mean ± SD values run in triplicate and repeated.

Cytotoxic effect of different chimeric DDS (with and without doxorubicin) was evaluated on the FR^+^ MCF7 cells using XTT assay^46^,^47^. The absorbance of resulting orange formazan derivative is measured spectrophotometrically at 450 nm 46. The toxicity of ^FA−^DDS^dox−^ (only capsid) and ^FA+^DDS^dox−^ (folic acid conjugated capsid) showed negligible effect on the viability up to tested concentration of delivery vehicles (from 0.55 μM to 4 μM) and incubation up to 48 hours. However, higher concentration of these DDS showed insignificant reduction (up to ~20%) in cell viability after 72 hours of incubation (Fig. 12a,b).

**Figure 13:**
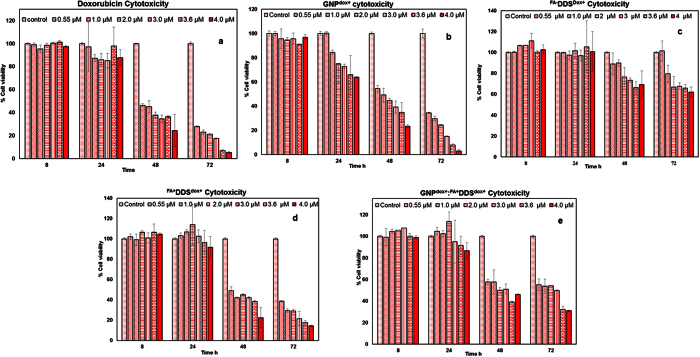
Cytotoxicity of various DDS formulations in MCF7 cells by XTT Assay: Cytotoxicity of various synthesized delivery vehicles containing selected equimolar doxorubicin such as (**a)** free doxorubicin (0.5 to 4 μM) (**b)** GNP^dox+^, (**c**) ^FA−^DDS^dox+^, (**d)** ^FA+^DDS^dox+^, and (**e)** GNP^dox+^:^FA+^DDS^dox+^ were evaluated on MCF7 cells for 8.0 h, 24, 48, and 72 hours. Cells were treated with calculated concentrations of these DDS (given along with each figure) and cell viability was measured in comparison to untreated control cells. ^FA−^DDS^dox+^ lacks targeting based internalization did not show any significant effect on cell viability. ^FA+^DDS^dox+^ and GNP^dox+^:^FA+^DDS^dox+^ showed the cytotoxicity after 48 hours but slower rate than equimolar doxorubicin containing GNP^dox+^ and free doxorubicin. The GNP^dox+^ and free doxorubicin showed higher cell toxicity due to rapid diffusion into the cells. Results were presented as mean ± SD values run in triplicate and repeated.

The cytotoxicity of doxorubicin conjugated modified delivery vehicles (GNP^dox+^,^FA−^DDS^dox+^, ^FA+^DDS^dox+^ and GNP^dox+^:^FA+^DDS ^dox+^) along with equimolar pure doxorubicin were evaluated on MCF7 cells (Table 2). 50–60% of cellular cytotoxicity was observed after 48 h and 80–90% after 72 h exposure depending upon the free doxorubicin concentration (0.55 μM to 4.0 μM) (Fig. 13a). However, pure doxorubicin showed very low cytotoxicity (~5–10%) up to 4.0 μM after 24 hrs. Similar results with little less cytotoxicity were observed with GNP^dox+^ containing equimolar concentration of doxorubicin (Fig. 13b).

The concentration of doxorubicin in ^FA+^DDS^dox+^ and GNP^dox+^:^FA+^DDS^dox+^ were equalized by concentration-based calculations/experiments (Table 2). The equimolar doxorubicin containing ^FA+^DDS^dox+^ and GNP^dox+^:^FA+^DDS^dox+^ results less cytotoxicity in comparison to free doxorubicin (Fig. 13d) while the ^FA−^DDS^dox+^ has more cell viability due to non-targeting nature. This is because uptaken delivery vehicles were first digested by lysosomal enzymes and then doxorubicin was released in cytoplasm. However, equimolar doxorubicin containing ^FA+^DDS^dox+^ showed higher cytotoxicity than GNP^dox+^: ^FA+^DDS^dox+^ of same drug concentration (Fig. 13e). The higher cytotoxicity of ^FA+^DDS^dox+^ was due to more release of doxorubicin after lysosomal digestion than GNP^dox+^:^FA+^DDS^dox+^. This higher release was due to use of 2.3 times higher concentration of ^FA+^DDS^dox+^ to equalize the total doxorubicin concentration in both drug delivery vehicles (Table 2). The doxorubicin associated with capsid were 2.3 time higher in ^FA+^DDS^dox+^ because other GNP^dox+^:^FA+^DDS^dox+^ contains approximately ~55.0% of its total doxorubicin associated with GNPs. This GNPs associated doxorubicin (as protected by the capsid in its core) was released only after complete lysosomal digestion of outer protein covering. The slow and multiple time persistent releases of doxorubicin form ^FA+^GNP-DDS^dox+^ can be advantageous to improve the efficacy of the system in animal model study. The cytotoxicity was gradually increased with time (after 48 and 72 h of incubation) and dose (from 0.5 to 4.0 μM). The order of cell cytotoxicity was higher in equimolar concentration of free doxorubicin > GNPs^dox+^ > ^FA+^DDS^dox+^ > GNP^dox+^:^FA+^DDS^dox+^ at all doxorubicin concentration. Free doxorubicin and doxorubicin conjugated to GNPs showed higher cytotoxicity (anticancer effect) because they quickly internalized in the cell through diffusion while internalization of doxorubicin bioconjugated with ^FA+^DDS^dox+^ was slow due to receptor mediated endocytosis^48^.

## Materials and Methods

### Materials

CCMV CP gene was chemically synthesized (Biobasic Inc India) after codon optimization. Specific PCR primers of truncated NΔ41, and NΔ26 CPs were procured from Eurofins, Bangalore India. HIS select cobalt resin, (1-Ethyl-3-(3-dimethylaminopropyl) carbodiimide (EDC), N-hydroxysuccinimide (NHS), Carboxyfluorescein succinimidyl ester (CFSE), doxorubicin, GNPs, α-Lipoic acid (α-LA) and XTT cytotoxicity kit were procured from Sigma Aldrich. Alexa fluor 610 NHS ester (AF610) was procured from life science technologies.

### Purification and characterization of N-terminal truncated capsid proteins

Full-length gene corresponding to CCMV capsid protein was chemically synthesized after *in silico* codon optimization. The gene was cloned into pET-21a expression vector having C-terminal his-tag with two variants of CP with N-terminal truncated sequences. These NΔ41 and NΔ26 CP variants were designated as F_1_ and F_2_ respectively. Both were overexpressed and purified using Co-NTA affinity purification and purity/molecular mass were confirmed SDS-PAGE and MALDI TOF. Structure function relationship was confirmed by intrinsic tryptophan fluorescence (ITF) (Scinco FS-2 fluorescence spectrometer) and far-ultraviolet (far UV) circular dichroism (CD) on JASCO J815 spectropolarimeter. The *in vitro* assembly of individual F_1_ and F_2_ as well as their combination in different molar ratio were performed by dialysis (MWCO 14 kDa, Sigma aldrich) in sodium acetate 0.1 M, sodium chloride 0.1 M, pH 4.8 for overnight at 4 °C. The *in vitro* assembly of individual F_1_ and F_2_ protein was also analyzed under purification conditions (with imidazole) and after purification and storage. The measurement of average particle mean diameter was performed on zeta sizer (Nano ZS 90, Malvern) equipped with a 5 mW helium/neon laser at 4 °C in triplicate. The results were expressed as average mean based on the number as variables for particle size distribution (PSD) in dynamic light scattering (DLS). The transmission electron microscope (FEI Tecnai G20) images were taken with 0.1% phosphotungstic acid (PTA). The mean diameter of assembled particles was calculated using Image J software. The pH stability of *in vitro* assembled VLPs was assessed at different pH from 4 to 8 overnight at 4 °C by zeta particle size analyzer and TEM.

### Bioconjugation of fluorescent dye

Carboxyfluorescein succinimidyl ester (CFSE) and Alexa fluor 610 NHS ester (AF610) were separately used to bioconjugate on F_1_ at 4 °C in 100 mM sodium phosphate pH 7.0 with constant stirring. Bioconjugation efficiency (number of dye molecule per CP) was controlled by molar ratio of dye/CP and by incubation time (1.0 min). Excess of reagents (dyes) and by-products (N-hydroxy succinimides) were removed by extensive dialysis in Tris-HCl pH 7.0. Fluorescent SDS-PAGE of unstained gel and coomassie brilliant blue (CBB) staining of same gel, fluorescence spectra (Excitation (Ex): 492 nm; Emission (Em): 520 nm for CFSE, and Ex: 610 nm; Em: 628 nm for AF610), absorbance (280 nm for CP; 492 nm for CFSE and 610 nm for AF610 dye) and HPLC quantification (initial solution and dialysate) were measured to confirm bioconjugation qualitatively and quantitatively.

### Bioconjugation of targeting ligand

Folic acid (22.6 mM, 78 μl) was activated with freshly prepared EDC (1645 mM, 102 μl) (10 times higher molar ratio), stabilized by NHS (1450 mM, 578 μl) (5 times higher molar ratio than EDC) for 30 min at room temperature and incubated with individual CP for bioconjugation in dark at 4 °C. The bioconjugation efficiency (number of folate molecule per CP) was controlled by maintaining various molar ratios of F_2_ and FA (1:10, 1:20, 1:50, 1:100, 1:200, 1:300). Reaction was terminated by removal of excess reactant and by-products by dialysis. The absorption (363 nm) and fluorescence spectra (Ex: 363 nm, Em: 450 nm) were used to determine the number of bioconjugated folic acid molecule per CP using work curve method as well as by HPLC quantification. The *in vitro* assembly potential of only FA bioconjugated CP (^FA+^F_2_) as well as in standard combination of molar ratio (F_1_: ^FA+^F_2_: 1:2) were confirmed by zeta particle size analysis and TEM imaging as described.

### Bioconjugation of doxorubicin

The amino group of doxorubicin (17.27 mM, 72.0 μl) was reacted with freshly prepared EDC-NHS (EDC = 197 mM, 25.35 μl and NHS = 1450 mM, 6.8 μl) and F_2_ (40 μM) in 2.0 ml at 4 °C overnight. Excess reactants were removed by dialysis and absorption (480 nm), fluorescence (Ex: 480 nm; Em: 592), HPLC quantification and SDS-PAGE (with and without CBB staining) were used to qualitatively and quantitatively determine the bioconjugation efficiency. The *in vitro* assembly of individual bioconjugated capsids (F_2_^dox+^) and in combination with F_1_ protein (F_1_: F_2_^dox+^: 1:2) was determined by zeta size analyzer and TEM.

### Synthesis of doxorubicin conjugated GNPs

The citrate stabilized GNPs of size ~10 nm were encapsidated by α-LA-Doxorubicin and 5′ thiolated RNA probe (CCCGACCTAGCCGACGAC) by overnight incubation at 4 °C. Briefly, 20 μl of α-Lipoic acid (1.0 M) and 16.26 μl of 5′ thiolated RNA probe (123 μM) were incubated overnight with 1.0 ml of GNPs (10 nM) at 4 °C in shaking condition and dialyzed. α-Lipoic acid was activated with 11.7 μl of EDC (1410 mM) and 25.2 μl of sulfo-NHS (1307 mM) for 30 min at room temperature. 10 μl of doxorubicin (0.55 mM) were further added and incubated overnight in shaking condition. Solution was dialyzed to remove excess molecules and byproduct to purify RNA-GNP-αLA-dox complex. Encapsulation and loading efficiency of 5′ thiolated RNA probe and α-Lipoic acid were determined using absorbance spectra. TEM, zeta size and potential analyses were performed to confirm the mono-dispersity, size and charge of GNP^dox+^ complex.

### Synthesis of chimeric drug delivery system

Chimeric drug delivery system (GNP^dox+^:^FA+^DDS^dox+^) was developed by *in vitro* assembly of three ligands bioconjugated CPs (DF_1_, ^FA+^F_2_, F_2_^dox+^) with GNP^dox+^ complex in its core. The assembly of chimeric delivery vehicles with GNP^dox^ was performed in a range of concentrations of different components of GNP^dox+^:^FA+^DDS^dox+^ for optimization. The final optimized concentrations of different components were, 100 μl GNP^dox+^ (42 μM), 100 μl of F_1_ (25 μM), 62.5 μl of F_2_ (56 μM), 70 μl of DF_1_ (25 μM), 25 μl of ^FA+^F_2_ (56 μM) and 62.5 μl of F_2_^dox+^ (56 μM). Final volume of these reactants was made 1.0 ml by adding phosphate buffer (100 mM, pH 7.0) and this solution was dialyzed overnight against assembly buffer. Assembly of chimeric delivery vehicles without GNPs^dox+^ was also performed in similar conditions (100 μl buffer in place of GNP^dox+^). The encapsidation of GNP^dox+^ was further confirmed without negative staining based TEM imaging and EDAX analysis. Zeta size analysis was performed to confirm the mono-dispersity and size of GNP^dox+^:^FA+^DDS^dox+^ and ^FA+^DDS^dox+^. The stability was confirmed by taking absorbance scan of DDS and GNP^dox+^:^FA+^DDS^dox+^ after incubation at 4 °C.

### *In vitro* release of drug

*In vitro* release experiment of doxorubicin from GNPs^dox+^, GNP^dox+^:^FA+^DDS^dox+^ and ^FA+^DDS^dox+^ were performed by dialysis method using 10.0 kDa cutoff dialysis membrane. Briefly, 1.0 ml of equimolar doxorubicin (~2.1 μM) GNP^dox+^, GNP^dox+^:^FA+^DDS^dox+^ and DDS^dox+^ along with lysosomal extract were placed in dialysis bag (10.0 kDa cutoff) and dialyzed against 20 ml phosphate buffer (100 mM) pH 7, at RT. The same experiments were repeated without the use of lysosomal extract and dialyzed against 20 ml phosphate buffer (100 mM) pH 7.2. Samples were collected at specific time point (0, 0.25 h, 0.5 h, 1, 2, 3, 4, 5, 6, 7, 8, 9 10, 11, 12, 22, 26, 28, 30, 32, 34, 36 so on till 83 h) from the dialysis buffer and doxorubicin was quantified by fluorescence measurement as described earlier. The experiment was performed thrice and the average values with standard deviation were reported.

### *In vitro* internalization assay

The FR^+^ MCF7 and FR^−^ HEK/HepG2 cells were grown/cultured in standard conditions using DMEM. The cells were grown till 50% confluence on coverslip and treated with 4.2 μM of each with ^FA+^DDS and ^FA−^DDS for 0, 5, 10, 15, 20, 30, 60 min at 37 °C. The cells were washed with PBS, fixed with chilled methanol and nuclear staining was carried out with Hoechst 3342 (1.0 μg/ml) and imaged on confocal laser scanning microscopy (LSM 710, Zeiss). For quantitative internalization assay, cells were grown in 3 mm dish till 50% confluence and treated separately with ^FA+^DDS and ^FA−^DDS for 0, 5, 10, 15, 20, 30 and 60 min. The cells were washed thrice with PBS and lysed with lysis buffer (50 mM Tris, 0.8% Triton, 0.2% SDS, pH 7.4). The relative intake was determined by fluorescence measurement of the lysate.

### Competitive inhibition assay

Media containing excess folic acid and ^FA+^DDS was added to the PBS washed MCF7 cells. After incubation for different time periods (0, 5, 10, 15, 20, 30, 60 min) the cells were washed thrice with PBS, fixed, nuclear stained with Hoechst 3342 and analyzed by confocal laser scanning microscopy for internalization of DDS.

### Cytotoxicity by XTT Assay

Cell viability assay was performed using XTT to evaluate the cytotoxic effect of different components of DDS such as free dox, ^FA+^DDS^dox−^ (no doxorubicin), ^FA−^DDS ^dox+^ (no targeting ability), ^FA+^DDS ^dox+^, GNP^dox+^:^FA+^DDS^dox+^ and GNP^dox+^ on MCF7 cells in 96 well plates. MCF7 cells were sub-cultured at a density of 5000 cells/well. At 50% confluence the culture medium was aspirated and cells were treated with different components of delivery vehicles (described above) in different concentrations (0.5, 1, 2, 3, 3.6 and 4 μM) for 4 h and washed. The cells were incubated for 8, 24, 48 and 72 h with 160 μl fresh media and 40 μl of XTT (1 mg/ml) (2,3-bis[2-Methoxy-4-nitro-5-sulfophenyl]-2H-tetrazolium-5-carboxyanilide inner salt) for 3 hours. The absorbance was measured at 450 nm with standard microplate reader (HybridSynergy2, Biogentek). The cell viability was calculated in comparison to the control cell.


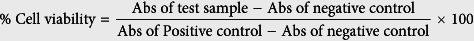


## Conclusion

This study demonstrates the development of targeted chimeric doxorubicin delivery nano-vehicles to FR overexpressed cancer cells (MCF7). Vehicle was generated through *in vitro* assembly of genetically engineered and ligands (dye, folic acid, dox) conjugated (EDC-NHS chemistry) capsid proteins of CCMV. The inner cavity of this vehicles were occupied by doxorubicin conjugated GNPs (10 nm) to increase the drug loading capacity and to improve drug efficacy. This vehicle showed the selective release of doxorubicin in cellular lysosome mimicking conditions during the *in vitro* release study. These vehicles showed the selective uptake and cytotoxicity in FR positive cells (MCF7) in comparison to FR negative cell lines (HepG2 &HEK) confirming the targeting potential of these vehicles.

## Additional Information

**How to cite this article**: Barwal, I. *et al*. Targeted delivery system for cancer cells consist of multiple ligands conjugated genetically modified CCMV capsid on doxorubicin GNPs complex. *Sci. Rep.* **6**, 37096; doi: 10.1038/srep37096 (2016).

**Publisher’s note:** Springer Nature remains neutral with regard to jurisdictional claims in published maps and institutional affiliations.

## Supplementary Material

Supplementary File

## Figures and Tables

**Table 1:** Complete detail of the concentration of native and ligands bioconjugated capsid proteins to synthesize different variants of chimeric delivery vehicles.

DDS Name	Constitutents detail	Final concentration of native and ligands bioconjugated capsid proteins (μM)					
		F_1_	DF_1_	F_2_	^FA+^F_2_	F_2_^dox+^	GNPs^dox+^
^FA+^DDS ^dox+^	DF_1_:^FA+^F_2_:F_2_^dox+^	2.5	1.7	1.68	3.36	3.36	—
GNPs^dox+^: ^FA+^DDS ^dox+^	DF_1_:^FA+^F_2_:F_2_^dox+^:GNPs^dox+^	2.5	1.7	1.68	3.36	3.36	0.067

**Table 2:** Corresponding concentrations of ^FA+^DDS^dox+^ and GNP^dox+^:^FA+^DDS^dox+^ containing equimolar doxorubicin concentration selected for the study.

S. No.	Conc. of doxorubicin selected for toxicity study (μM)	Equimolar Conc. of ^FA+^DDS^dox+^ (μM)	Equimolar Concn. of GNP ^dox+^-^FA+^DDS^dox+^ (μM)
1	0.5	0.038	0.016
2	1.0	0.070	0.030
3	2.0	0.190	0.060
4	3.0	0.210	0.090
5	3.6	0.252	0.108
6	4.0	0.280	0.120
