# Supplementary material for: Targeted delivery system for cancer cells consist of multiple ligands conjugated genetically modified CCMV capsid on doxorubicin GNPs complex
Source: Sci Rep. 2016 Nov 22;6:37096. doi: 10.1038/srep37096 (PMC5118717; doi:10.1038/srep37096)
Supplement: Supplementary File [file srep37096-s1.pdf]

Targeted delivery system for cancer cells consist of multiple ligands conjugated genetically modified CCMV capsid on doxorubicin GNPs complex

Indu Barwal[12], Rajiv Kumar[3], Suneel Kateriya[4], Amit Kumar Dinda[5], Subhash Chandra Yadav[26]*

[1]TERI University, Vasant Kunj, New Delhi-110070, India.

[2]TERI-Deakin Nano Biotechnology Centre, The Energy and Resources Institute, Darbari Seth Block, IHC Complex, Lodhi Road, New Delhi – 110003, India.

[3]School of Molecular Medicine, Jawaharlal Nehru University, New Delhi

[4]School of Biotechnology, Jawaharlal Nehru University, New Delhi

[5]Department of Pathology, All India Institute of Medical Sciences, New Delhi 110029

[6]Department of Anatomy, All India Institute of Medical Sciences, New Delhi 110029

*Corresponding Authors

Corresponding author address:

TERI-Deakin Nano Biotechnology Centre, The Energy and Resources Institute, Darbari Seth Block, IHC Complex, Lodhi Road, New Delhi – 110003, India. Contact no: 91-11-24682100, ext. 2610; mobile: +919868126177; Fax: 91-11 24682144; Email: subhashmbu@gmail.com

Present address: Nanobiotechnology Lab, Convergence Block Basement, Electron Microscope Facility, Department of Anatomy, All India Institute of Medical Sciences (AIIMS), New Delhi- 110029, India, Contact No. 919868126177; Email: subhashmbu@gmail.com

<u>Supplementary Figure 1:</u> Cloning of complete gene

For the bulk production of CCMV capsid protein in *E. coli* system, codon optimization was a necessary step to achieve overexpression of protein because many of viral codons were rarely used (low usage codons) or having expression-limiting regulatory elements in *E. coli* systems[1]. For the cloning and protein engineering, DNA and protein sequence of CCMV capsid protein were taken from NCBI (NC_003542.1) and PDB (1cwp) respectively[2,3]. The codon optimization was done by codon optimization software (https://eu.idtdna.com/CodonOpt). By using the genome of natural CCMV virus through PCR amplification of capsid gene, it was very difficult to accomplish codon optimization. Thus, the complete gene of capsid protein was chemically synthesized after codon optimization (Supplementary Figure 1a). Two restriction sites BamHI and XhoI were *in silico* added in the artificially synthesized gene frame. These RE sites were introduced considering the use of pET21a vector for expression. These sites add minimum cloning based extra N-terminal (14) and C-terminal (8) amino acid sequences (Supplementary Figure 1b).

```
NCBI-Sequence   ------atgtctacagtcggaacagggaagttaactcgtgcacaacgaagggctgcggcc
Codon-Optimized ggatccatgtctaccgtgggtaccggtaaactgactcgtgctcaacgtcgtgctgccgca
                      ********.** **:**.** **. *.********:*****:.* ***** **.
NCBI-Sequence   cgtaaaaacaagcggaacactcgtgtggtccaacctgttattgtaaaacccatcgcttca
Codon-Optimized cgtaaaaataagcgcaacacccgcgtggtccagccagtaattgtggaaccgatcgcatcc
                ******** ***** ***** ** ********.**:**:*****..**** *****:**.
NCBI-Sequence   ggccaaggcaaggctattaaagcgtgggccggttacagcgtatcgaagtggaccgcctca
Codon-Optimized ggtcagggcaaagcgatcaaagcatggaccggttattctgtttctaaatggacggcctcc
                ** **.*****.** ** *****.***.*******  :   **:** **.***** *****.
NCBI-Sequence   tgtgcggctgccgaagctaaagtaacctcggctataactatttctcttcctaacgagcta
Codon-Optimized tgtgcggccgcggaggcgaaggttacttccgcgatcactatcagcctgcctaacgaactg
                ******** ** **.** **.**:** ** ** **.***** :  ** ********.**.
NCBI-Sequence   tcatccgaaaggaataaacagctcaaagtggggagagttttattatggcttgggttgctt
Codon-Optimized tcttccgaacgcaacaagcagctgaaagtaggtcgtgttctgctgtggctgggtctgctg
                **:******.* ** **.***** *****.** .*:*** *. *.***** **  ****
NCBI-Sequence   cccagtgtcagtggtacggtgaaatcctgtgtcacagagacgcagactacagctgctgct
Codon-Optimized ccgagcgtaagcggcactgttaaaagctgcgttacggaaactcagactaccgcagctgca
                ** ** **.** ** ** ***: *** ** **.**.** ********.**:*****:
NCBI-Sequence   tcctttcaggtggcattagccgtggccgacaactcgaaagatgttgtcgctgctttgtac
Codon-Optimized tctttccaggtggcactggctgtagcggacaactctaaagacgtggtggccgctatgtac
                ** ** ********* *.** **.** ******** ***** ** ** ** ***:*****
NCBI-Sequence   cccgaggcgtttaagggcataacccttgaacaactcgctgcggatttaacgatctacttg
Codon-Optimized ccggaggcttttaaaggcattaccctggaacagctggcggcggatctgaccatctatctg
                ** ***** *****.*****:***** *****.** ** ****** *.** *****  **
NCBI-Sequence   tacagcagtgcggctctcactgagggcgacgtcatcgtgcatttggaggttgagcacgtc
Codon-Optimized tactcttccgctgcgctgaccgaaggcgatgttattgtccatctggaagtcgaacacgtt
                 ***:   :  ** ** ** ** **.***** ** ** ** *** ****.** **.*****
NCBI-Sequence    agacctacgtttgacgactcgttcactccggtgtat------
Codon-Optimized  cgtccgaccttcgatgactccttcaccccggtttacctcgag
                  .*:** ** ** ** ***** ***** ***** **
```

Supplementary Figure 1a: Clustal omega alignment of NCBI gene sequence (derived from 1360 to 1932 gene sequences of NCBI id NP_613277.1) and codon optimized (for *E. coli*) capsid gene of CCMV. The restriction sites **ggatcc** for BamHI (N-terminal) and **ctcgag** for XhoI (C-terminal) were added in codon-optimized gene.

```
PDB-Sequence          --------------MSTVGTGKLTRAQRRAAARKNKRNTRVVQPVIVEPIASGQGKAIKA
In-silico-Sequence    MASMTGGQQMGRGSMSTVGTGKLTRAQRRAAARKNKRNTRVVQPVIVEPIASGQGKAIKA
                                    **********************************************
PDB-Sequence          WTGYSVSKWTASCAAAEAKVTSAITISLPNELSSERNKQLKVGRVLLWLGLLPSVSGTVK
In-silico-Sequence    WTGYSVSKWTASCAAAEAKVTSAITISLPNELSSERNKQLKVGRVLLWLGLLPSVSGTVK
                      ************************************************************
PDB-Sequence          SCVTETQTTAAASFQVALAVADNSKDVVAAMYPEAFKGITLEQLTADLTIYLYSSALTE
In-silico-Sequence    SCVTETQTTAAASFQVALAVADNSKDVVAAMYPEAFKGITLEQLTADLTIYLYSSALTE
                      ************************************************************
PDB-Sequence          GDVIVHLEVEHVRPTFDDSFTPVY--------
In-silico-Sequence    GDVIVHLEVEHVRPTFDDSFTPVYLEHHHHHH
                      ************************
```

Supplementary Figure 1b: Clustal omega alignment of CCMV capsid protein sequence (PDB id 1cwp) and *in silico* protein sequence generated from the codon optimized gene sequence (*in-silico* sequence). N-terminal 14 amino acid (**MASMTGGQQMGRGS**) and C-terminal 8.0 amino acid (**LEHHHHHH**) were represented in ***in-silico*** protein **sequence** to be expressed by BamHI and XhoI restriction enzyme based cloning in pET-21a plasmids.

Supplementary Figure 2: Optimization of N terminal truncation

Methods: Numbers of N-terminal truncations (NΔ10, NΔ26, NΔ35, NΔ41, NΔ51) were generated *in silico* based on the crystallographic structure and functionality of the sequences[3,4]. These sequences were submitted to I-TASSER and GalaxyGemini tool for the structural conformation (structure function relationship) and assembling potential respectively. Based on the outcome of these analysis, the NΔ41 and NΔ26 truncated capsid protein were selected for development of drug delivery vehicles. These NΔ41 and NΔ26 capsid protein variants were designated as F_1 and F_2 respectively.

Results and Discussion: CCMV capsid is well-studied and first i*n vitro* assembled capsid from individual components. Protein-protein and RNA interaction play an important role in capsid formation. The N-terminus sequence was employed for identifying and binding the negatively charged RNA strands while the C terminus sequence takes part in inter-subunit linking. Hence the role of N-terminus was mainly to recognize and load RNA molecule in icosahedral capsid. However, these sequences were not required for *in vitro* assembly. Hence, these N-terminus sequences could be deleted without affecting the assembly *in vitro*[4]. Two variants of N-terminal truncated sequences were reported by deleting first 26 and 41 amino acid of capsid protein[4]. The deletion of these amino acid sequences completely blocked the RNA mediated assembly but had no effect on normal assembly[5]. The 27-35 N-terminal amino acid sequences form β-hexamer responsible for providing the stability to hexameric capsomers. This enhances the *in vitro* assembly through trimer formation in suitable condition[3]. However, amino acid sequence 44-51 forms the clamps that hold the C-terminal sequences of other protein. This is responsible

for formation of non-covalent dimer, which finally lead to formation of aggregates/assembly depending upon solvent composition[3,6,7]. Thus to achieve efficient virion formation in assembly condition only and also to avoid assembly during overexpression/ purification, two variants of N-terminal truncated capsid protein NΔ41 (F_1) and NΔ26 (F_2) were proposed to synthesize by recombinant DNA technology based expression. To generate NΔ41 (F_1) and NΔ26 (F_2), the capsid protein gene were PCR amplified using specific primers having extra sequence for introduction of BamHI (in both forward primers) and XhoI (reverse primer) (Supplementary Table 1).

Supplementary Table 1: PCR primers to amplify N-terminus truncated NΔ41 (F_1) and NΔ26 (F_2) capsid protein gene

**Protein**	**Primers**	**Sequence**
**F_1**	Forward primer 1	GTC **GGA TCC** AAA GCG ATC AAA GCA TGG ACC
**F_2**	Forward primer 2	GTC **GGA TCC** GTG GTC CAG CCA GTA ATT GTG
**F_1 and F_2**	Reverse primer	ATC **CTC GAG** GTA AAC CGG GGT GAA GGA GTC

PCR amplification using the full-length capsid protein gene as template and specific primers leads to generation of N-terminus deletion variants of capsid proteins (Supplementary Figure 2a). The N terminus deletion mutants were modelled and analyzed *in silico* for their ability to oligomerize and to form the capsid. The analysis involves the generation of 3D atomic models from iterative structural assembly simulation and multiple threading alignments followed by homology modeling[8].

```
In-silico-Sequence MASMTGGQQMGRGSMSTVGTGKLTRAQRRAAARKNKRNTRVVQPVIVEPIASGQGKAIKA
F1-NΔ41            MASMTGGQQMGRGS-----------------------------------------KAIKA
F2-NΔ26            MASMTGGQQMGRGS--------------------------VVQPVIVEPIASGQGKAIKA
                   **************                                         *****

In-silico-Sequence WTGYSVSKWTASCAAAEAKVTSAITISLPNELSSERNKQLKVGRVLLWLGLLPSVSGTVK
F1-NΔ41            WTGYSVSKWTASCAAAEAKVTSAITISLPNELSSERNKQLKVGRVLLWLGLLPSVSGTVK
F2-NΔ26            WTGYSVSKWTASCAAAEAKVTSAITISLPNELSSERNKQLKVGRVLLWLGLLPSVSGTVK
                   ************************************************************

In-silico-Sequence SCVTETQTTAAASFQVALAVADNSKDVVAAMYPEAFKGITLEQLTADLTIYLYSSAALTE
F1-NΔ41            SCVTETQTTAAASFQVALAVADNSKDVVAAMYPEAFKGITLEQLTADLTIYLYSSAALTE
F2-NΔ26            SCVTETQTTAAASFQVALAVADNSKDVVAAMYPEAFKGITLEQLTADLTIYLYSSAALTE
                   ************************************************************

In-silico-Sequence GDVIVHLEVEHVRPTFDDSFTPVYLEHHHHHH
F1-NΔ41            GDVIVHLEVEHVRPTFDDSFTPVYLEHHHHHH
F2-NΔ26            GDVIVHLEVEHVRPTFDDSFTPVYLEHHHHHH
                   ********************************
```

Supplementary Figure 2a: Alignment of completely overexpressed amino acid sequence of full protein (*in silico* sequence), F_1 (NΔ41) and F_2 (NΔ26) in *E. coli* using pET-21a cloning strategies. The cloning based N- and C- terminal extra amino acid sequences were given in red and blue respectively.

The results from I-TASSER confirmed the computational integrity of folding pattern with the additional sequences of N- and C-terminal (Supplementary Figure 2b). The property of N-terminal truncated capsid proteins F_1 and F_2 to form trimeric T3 symmetry based assembly remained intact after sequence modification. This was confirmed by Galaxy Web computational tool (http://galaxy.seoklab.org/cgi-bin/submit.cgi?type=GEMINI) (Supplementary Figure 2c). This tool predicts the oligomerization pattern of proteins at molecular level based on the tertiary structure[9]. Both the analysis confirmed the modified capsid proteins were viable for folding and assembly into capsid.

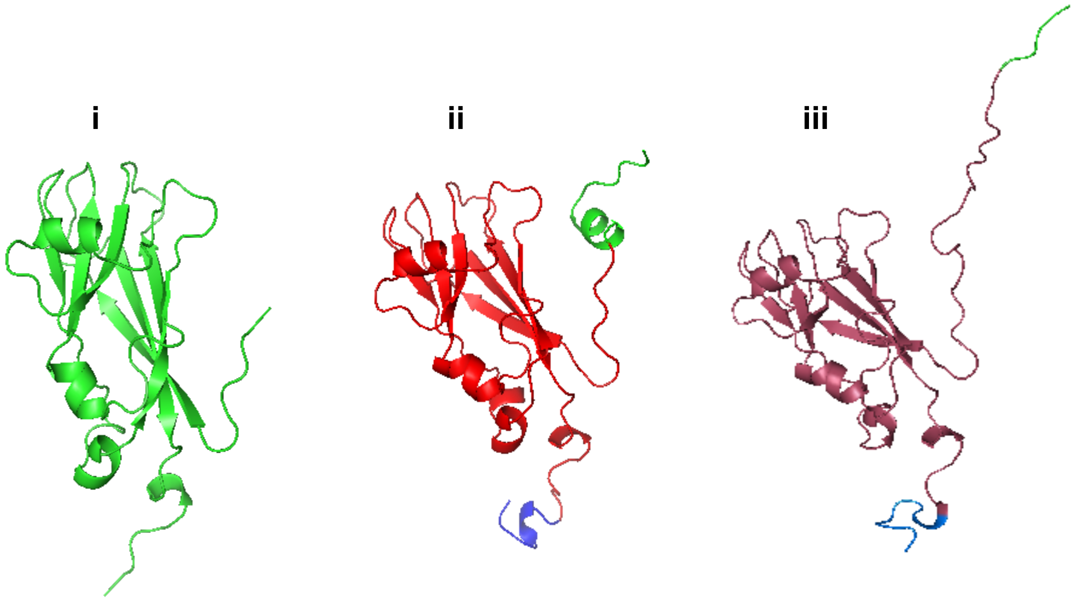

Supplementary Figure 2b: Conformational structure of the modified CCMV capsid protein modeled by I-TASSER bioinformatics tool. (i) CCMV capsid protein from PDB (PDB id 1cwp) **(ii)** for F_1 (NΔ41) and **(iii)** F_2 (NΔ26). The comparative structural study revealed that both the modified protein has nearly same structure as their original pdb structure. The cloning based additional sequence does not affect the structure function relationship *in silico*. The additional N-terminal and C-terminal protein sequence were represented in green and blue color respectively.

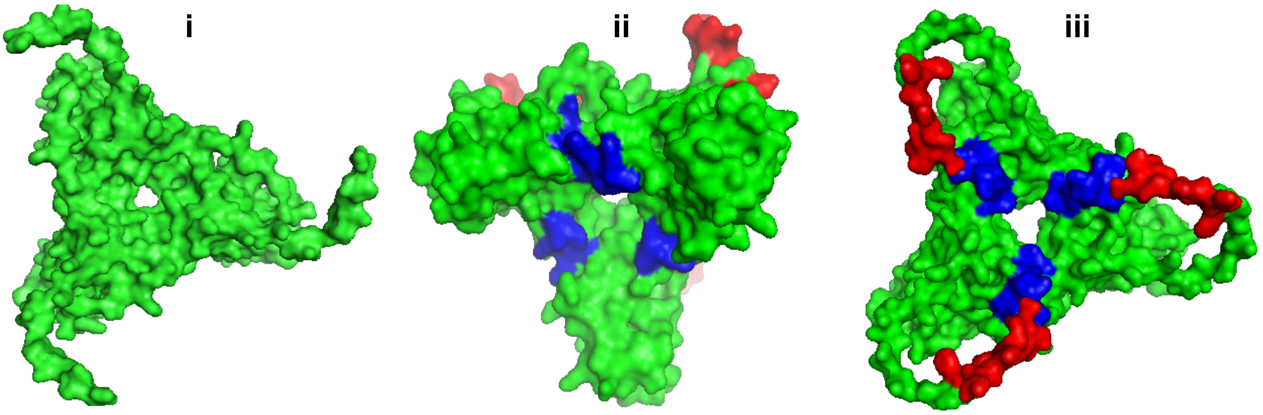

Supplementary Figure 2c: *In silico* analysis of assembling ability using GalaxyGEMINI tool of **(i)** Native **(ii)** F_1 (NΔ41) and **(iii)** F_2 (NΔ26) capsid proteins. The N terminus additional 14 amino acid sequences (by cloning strategies) were shown in red while the C terminus his-tag was shown in blue color.

Supplementary Figure 3: Cloning of F_1 and F_2 gene to cloning vector

Methods: The codon-optimized gene was received as pUC57 construct having BamHI and XhoI restriction site at 5′ and 3′ end of the gene. It was dissolved in autoclaved millique water to prepare 1.0 μg/ml stock solution, which was further diluted as 0.01 μg/ml as working solution. This vector was amplified in *E. coli.* (DH5α) and restriction digestion using BamHI and XhoI was performed. Briefly; the reaction was performed at 37˚C for 2 h incubation using the Neb buffer 4 (1X), BSA (1X), BamHI (1μl) and XhoI (1μl) were used for 50 μl reaction to digest ~1 μg DNA. The digested product along with DNA marker and undigested plasmid were electrophoresed on 0.8% agarose gel containing ethidium bromide in TE buffer. The fragment was visualized on UV illumination using FluorChem M AlphaImager (Protein Simple, USA).

The full-length pUC-57 gene construct was used as template to amplify the F_1 (459bp) and F_2 (504bp) gene by PCR. The PCR primer (Eurofins Genomics, USA) containing restriction sites (BamHI (GGATCC) in forward and XhoI (CTCGAG) in reverse primer) as flanking region was designed by using Primer3 software for PCR amplification of both F_1 and F_2 capsid protein gene (Table 1) (http://primer3.ut.ee/). The F_1 (459bp) and F_2 (504bp) capsid gene were PCR amplified. The PCR reaction was set for 25 μl using final 1X PCR buffer, 1.5 mM $MgCl_2$, 0.2 mM dNTPs, 0.2 μM each of forward and backward primers, 1U of Taq polymerase and 50 ng of template DNA. The programming of PCR (Biorad, USA) (30 cycles) were heating at 95°C for 4 min, denaturation at 94°C for 1 min, annealing at 57°C (for F_1)/ 58°C (for F_2) for 30 seconds and extension at 72°C for 1 min. The amplified PCR product for F_1 and F_2 were extracted and purified using gel extraction kit (mdi membrane technologies, India) as per the manufacturer's protocol. The eluted product was quantified using microplate reader (HybridSynergy2, BioTek, USA).

The purified PCR products for F_1 and F_2 were ligated into pGEM-T Easy vector (Promega, USA) by TA cloning using 3:1 molar ratio of PCR product and vector respectively. The ligation reaction (10 μl) of amplified capsid inserts and pGEM-T Easy vector was catalyzed by T4 DNA Ligase (5 units/ μl) in presence of 1X ligation buffer at 16˚C overnight. The gene constructs after ligation into pGEM-T vector were transformed into competent *E.coli* (DH 5α) cells by using heat shock (42°C) method. The cells were grown on LA plate containing ampicillin as selection markers. Colony PCR was done to screen the positive clones containing the gene of interest. The positive clones from single colony were amplified in LB medium containing ampicillin. The plasmids were isolated

and cloning was confirmed by restriction digestion. The positive constructs were further sequenced for confirmation. The pGEM-T vectors constructs of F_1 and F_2 protein cloned into pET-21a vector (using BamHI and XhoI). The positive constructs were transformed in *E.coli* BL21 competent cells, selected by ampicillin, glycerol stocks were prepared and stored at -80ºC (Supplementary Figure 3a).

MALDI TOF of Intact protein

Mass spectrometry (MS) was used to determine the molecular mass of F1 and F_2 capsid protein just after the affinity purification. The proteins solutions were extensively dialyzed against Millipore water and 0.8 µl of the sample was spotted on a MALDI plate. Then 1 µl of sinapinic acid was used as matrix after proteins samples were dried. Multiple standard were used for the calibration of the equipment. The data were obtained in MALDI-TOF TOF 4800 using linear spectrum.

Results and Discussion: The artificially synthesized full capsid gene in pUC57 vector were amplified and confirmed by restriction digestion. The restriction digestion product of 582 bp confirmed the cloned gene of interest (Supplementary Figure 3b-i). The desired

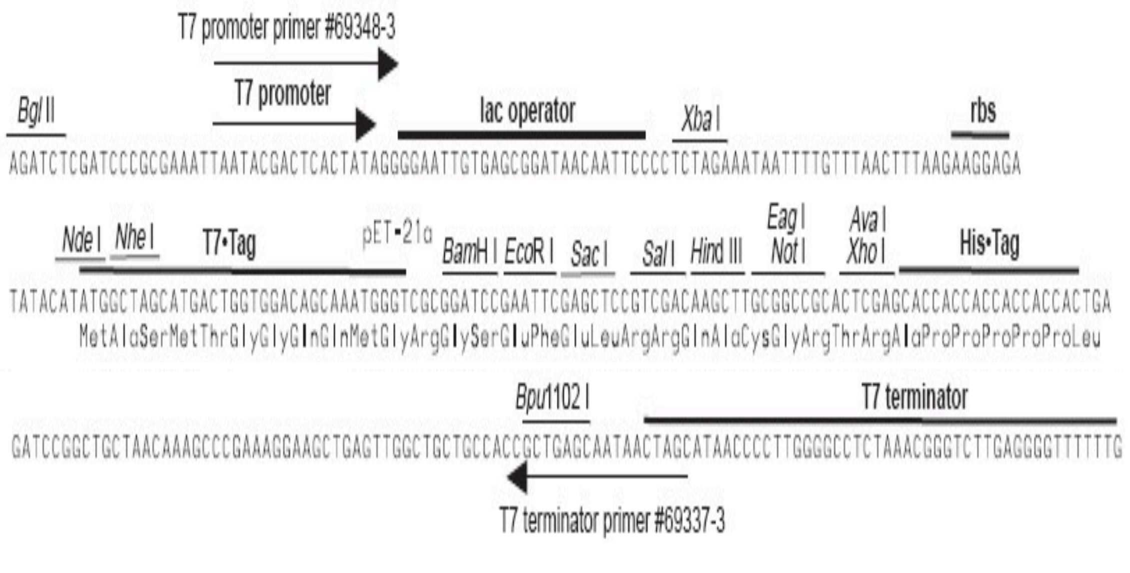

Supplementary Figure 3a: **pET21a vector map detail for promoter and multiple cloning sites (MCS).** The BamH I and Xho I were the first and last restriction sites in the MCS of pET-21a vector respectively. Cloning of any gene between these sites add only 14 amino acid (MASMTGGQQMGRGS) at N-terminal and 8 amino acid including his-tag at C-terminal (LEHHHHHH).

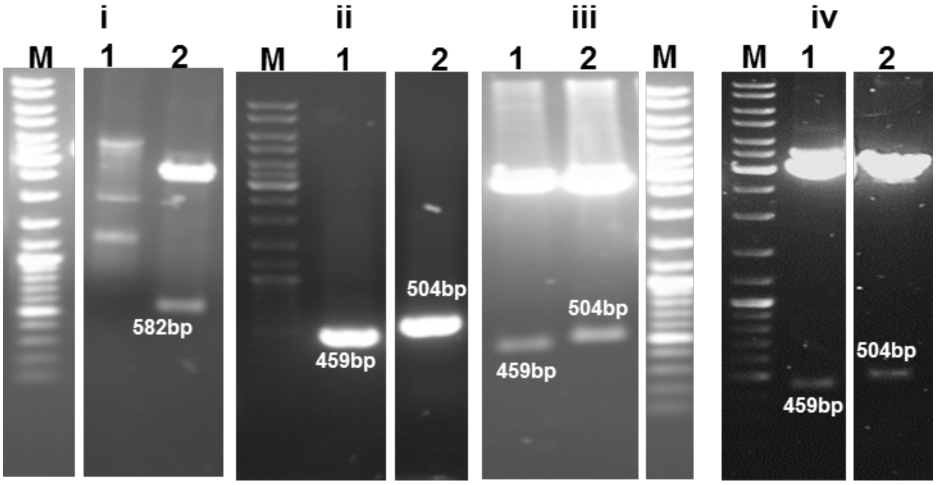

Supplementary Figure 3b: Cloning and confirmation of F_1 and F_2 genes in intermediary (pGEM-T) and expression vector (pET-21a). (i) Double digestion of pUC57 cloning vector using BamHI and XhoI restriction enzymes (lane 1: undigested plasmid, lane 2: digested plasmids, M: 1kb ladder). **(ii)** PCR amplification of F_1 and F_2 genes sequences using the primers given in table 1 (lane 1: F_1, lane 2: F_2, and M: 1kb ladder). **(iii)** Double digestion of pGEM-T intermediary vector (lane 1; digested plasmid of F_1, lane 2; digested plasmid of F_2, and M; 1kb ladder). **(iv)** Double digestion of pET-21a expression vector (M; 1kb ladder, lane 1; digested plasmid of F_1, lane 2; digested plasmid of F_2). The product size for F_1 and F_2 were 459bp and 504bp respectively.

sequences of F_1 and F_2 protein gene were PCR amplified using primers given in the Table 1 (Supplementary Figure 3b-ii). These primers added the desired restriction sites at 5′ and 3′ end for the new genes. The PCR amplified genes of interest (F_1 and F_2) was cloned in to an intermediary vector pGEM-T using TA cloning. The cloned pGEM-T vectors were further amplified and the cloning of these genes were confirmed by restriction digestion using BamHI and XhoI. The pGEM-T constructs of 459 bp (F_1) and 504 bp (F_2) were sequenced to confirm the desired sequence of F_1 and F_2 genes.

This cloning helps to improve the cloning efficiency than direct cloning into expression vector pET21a from PCR product. The current cloning strategies was used to add minimum amino acids on N- and C-terminals of each capsid proteins (Supplementary Figure 3a). This is because the capsids proteins need to be *in vitro* assembled to form the capsid by oligomerization and these additional sequences may have adverse effect on formation of stable drug delivery vehicles. The pGEM-T constructs were cloned into pET-21a for bulk expression in *E. coli*. The computational analysis of protein demonstrated that the addition of sequences at N-terminus and his-tag at C-terminal derived from pET-21a vector on each capsid protein had minimal effect with respect to folding and structure function relationship[10]. Thus, these gene sequences were cloned into pET-21a vector that characteristically introduced his-tag at C-terminus for ease in purification[11]. pET-21a

vector is based on promoter from T7 phage RNA polymerase and requires a host strain with DE3 phage fragment as lysogen, which encodes the T7 RNA polymerase (bacteriophage T7 gene 1) under the control of the IPTG inducible lacUV5 promoter for protein expression (Supplementary Figure 3a). The expression of the recombinant protein using these plasmids was tightly regulated by repression of the lacUV5 promoter by LacI and produces high levels of transcripts and recombinant proteins on induction by IPTG[12]. T7 RNA polymerase is transcribed when IPTG binds and triggers the release of repressor from the lac operator. The pET-21a construct of modified capsid proteins (F_1 and F_2) were confirmed by double restriction digestion (Figure 3b-iv).

The F_1 and F_2 gene construct were cloned into expression vector pET-21a with BamHI and XhoI sites. Usage of BamHI and XhoI restriction enzymes added 14 amino acids at N terminus (MASMTGGQQMGRGS) and eight amino acid (LEHHHHHH) at C-terminus. These additions did not affect the folding and assembly of the capsid. The effect of the additional amino acid residues on folding and possible effect on structure was evaluated using I-TASSER[8]. This prediction tool was used for analyzing the effect of modified sequence to structure-function paradigm. Its prediction are highly reliable because it first generates 3D atomic models from iterative structural assembly simulation and multiple threading alignments followed by homology modeling[8]. The property of N-terminal truncated capsid proteins F_1 and F_2 to form trimeric T=3 symmetry based assembly remained intact after sequence modification. This was also confirmed by Galaxy Web computational tool (http://galaxy.seoklab.org/cgi-bin/submit.cgi?type=GEMINI) (Supplementary figure 2). This tool predicts the oligomerization pattern of proteins at molecular level based on the tertiary structure[9].

Overexpression and purification of F_1 and F_2 capsid protein

The solubility predictions upon overexpression in *E.coli* were analyzed using SOLpro tool (http://scratch.proteomics.ics.uci.edu) for both F_1 and F_2 capsid protein of CCMV. This tool indicated nearly 52% insoluble probability for F_1 and very high probability (~60%) for solubility of F_2 protein[13]. Based on the results, the primary culture (*E. coli* BL21) were grown at 37°C overnight with continuous shaking at 150 rpm. This culture (10.0 ml) was transformed in 1 liter sterilized terrific broth medium (TBM) containing ampicillin (100µg/ml) and grown at 37ºC with continuous shaking (150 rpm) till the culture OD_{600} reaches 0.4-0.6. The complete culture was cooled on ice and induced for expression by 0.3mM IPTG. The cultures were further grown at 16ºC for 48h in continuous shaking condition. The cells were harvested by centrifugation (7000g) for 10

min at 4ºC. The harvested cells were lysed by sonication for 3 min (40% amplitude, for 2 sec pulse and 5 sec relaxation, in ice) to extract the protein. The cell debris was removed by centrifugation (14k rpm) for 15 min at 4ºC and the soluble fraction was filter sterilized and stored at 4ºC for further use.

HIS-select cobalt affinity gel (Sigma Aldrich, USA) was used for immobilized metal ion affinity chromatography (IMAC). The affinity column was washed with 5-column volume of buffer and equilibrated with chilled 1X PBS buffer. The overexpressed capsid proteins (F_1 and F_2) were allowed to bind pre-equilibrated cobalt-NTA beads separately and washed using 1XPBS and 20mM imidazole to remove non-specific bound proteins. His-tagged capsid protein was eluted using 250mM imidazole prepared in 1XPBS buffer.

Supplementary Figure 4: Western blot

The affinity purified capsid proteins were visualized in 15% SDS-PAGE gel[14]. His-tagged CCMV capsid protein variants were confirmed by western blotting using anti-his antibody (from mouse). The blots were transferred to nitrocellulose membrane at constant voltage (100V) for an hour.

The membrane was blocked using 5% dried milk in 1X PBS for 1h at 4°C with continuous shaking. This membrane was washed thrice with 1X PBS with 0.1% Tween20. The washed membrane was incubated with primary antibody (diluted 1:1000) containing 1% BSA in 1X PBS for 1 hour at room temperature. This membrane was again washed thrice to remove the excess antibodies and incubated with secondary antibody (Horse radish peroxidase (HRP)-conjugated rabbit anti-mouse IgG) at 1:8000 dilutions for 1 hour at room temperature. The membrane was washed five times and the blot was developed in dark on X-ray film by chemiluminescence produced due to action of HRP on luminol (dissolved in DMSO) with p-coumaric acid (dissolved in DMSO) and hydrogen peroxide. These antibodies bind to the C-terminal his-tag of each capsid proteins (F_1 and F_2). The HRP conjugated rabbit anti-mouse secondary antibody was used to bind and produce the signal by the conversion of luminol to 3-aminophthalate in presence of hydrogen peroxide. The signal was enhanced by chemiluminescence by using p-coumaric acid (Supplementary Figure 4).

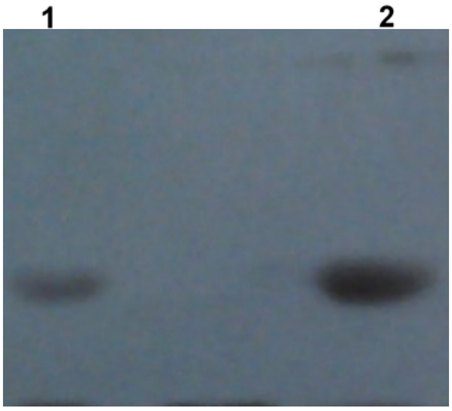

Supplementary Figure 4 : Western blot analysis of his-tagged CCMV capsid proteins. Lane 1; F_1 and lane 2: F_2 capsid protein. This was done using mouse anti-his IgG to indirectly identify the overexpression of capsid proteins by confirming his-tag expression. Mouse anti-his monoclonal antibody (dilution: 1:1000) and anti-mouse HRP conjugated antibody (dilution: 1:8000) were used as primary and secondary antibody for the western blotting.

Supplementary Figure 5: Biophysical characterization

Intrinsic tryptophan fluorescence

For intrinsic tryptophan fluorescence, the purified protein was diluted in phosphate buffer to a final concentration of 0.01mg/ml. Analysis of the protein solution was performed in a fluorimeter (Scinco FS-2 fluorescence spectrometer, Korea) using a quartz cell with 1 cm as path length. Spectra were taken by protein excitation at 292 nm and the fluorescence was measured at 300 to 400 nm. Similarly the fluorescence of modified

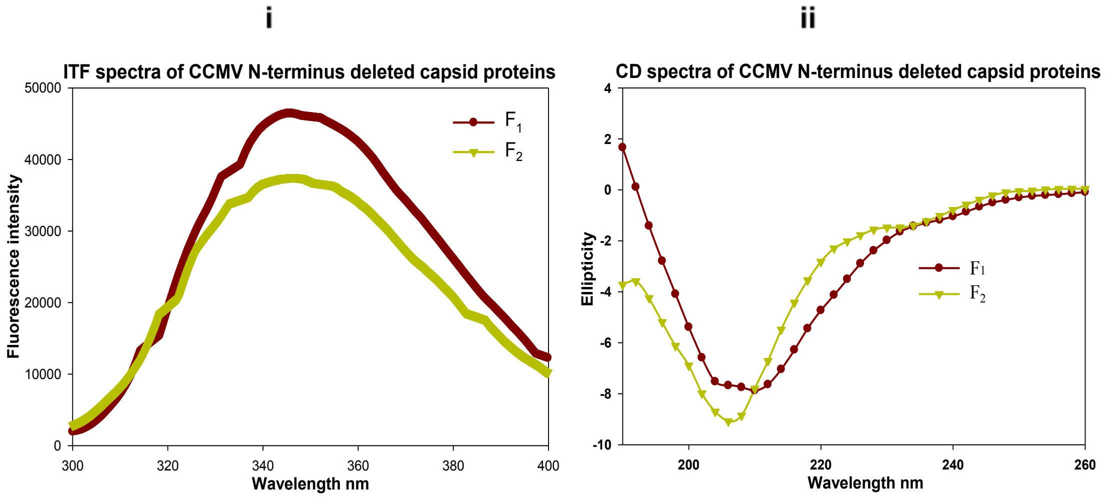

Supplementary Figure 5a: Biophysical characterization by (i) intrinsic tryptophan fluorescence (ITF) and (ii) Far UV CD. For ITF measurements the 0.01mg/ml of F_1 and F_2 capsid proteins were excited at 292 nm and emission was recorded from 300 to 400 nm. The emission spectra for the capsid proteins were found near 340nm, which confirmed the folded state of proteins. The Far UV CD spectra of F_1 and F_2 capsid proteins (0.10mg/ml) were taken from 190 to 260nm at ambient temperature. The CD spectra with the negative ellipticity at 208 nm and positive peak below 200nm suggested the folded structure of the proteins.

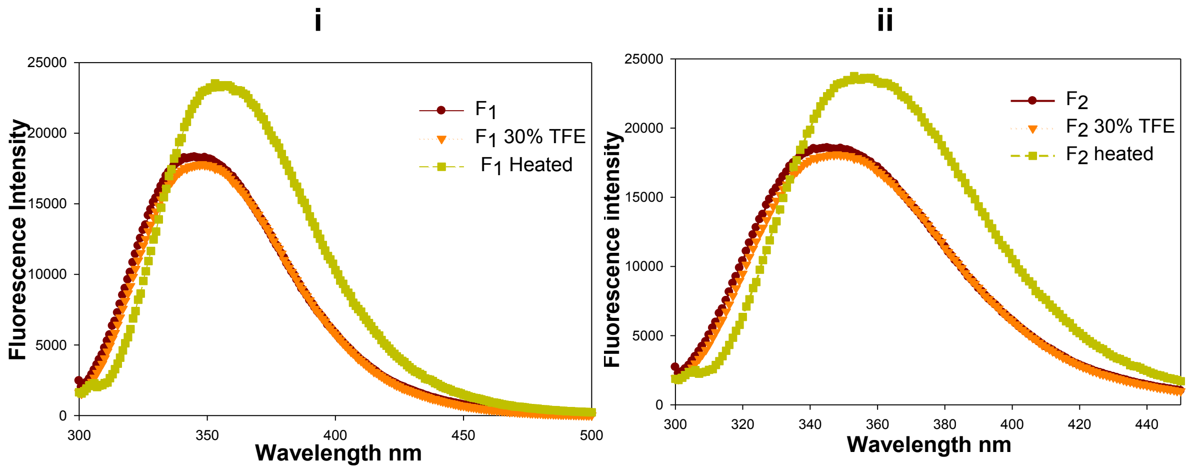

Supplementary Figure 5b: ITF of (i) F_1 and (ii) F_2 capsid proteins in different conditions. The intrinsic tryptophan fluorescence of 0.01mg/ml capsid protein was measured in native (phosphate buffer), with 30% TFE and heat-denatured conditions (95°C). The protein solutions were excited at 292 nm and emission spectra was recorded between 300 to 400 nm. The native folded protein in phosphate buffer and TFE showed emission at 340 nm while heat induced denatured at 355 nm due to exposure of buried tryptophan residues.

capsid protein (F_1 and F_2) was measured in different conditions *e.g.* in presence of trifluoroethanol (30% TFE) and denaturing conditions (heat 95°C, 10 min) to see the effect on folding of both proteins.

Far UV circular dichroism (CD) spectrum

The capsid protein sample was diluted in low ionic strength phosphate buffer, pH 7 (50mM) to a final concentration of 0.1mg/ml. The protein was analyzed in a CD spectrometer (JASCO J-815 spectropolarimeter, Japan) using a quartz cell with a path length 1 mm. Spectra was recorded with 1.0 nm bandwidth over the wavelength range of 190 nm to 260 nm. The final spectra was averaged from 3 scans and subtracted from the solvent spectra. Ellipticity values of CD spectra were expressed as the mean residue ellipticity (Θ).

Supplementary Figure 6: *In vitro* assembly of CCMV capsid

The assembly of individual F_1 and F_2 capsid protein were analyzed under purification conditions (with imidazole) as well as after storage up to one month in purified form. The capsid formation by *in vitro* assembly of individual F_1 and F_2 as well as their combination in different molar ratio was performed by dialysis (MWCO 14 kDa, Sigma Aldrich, USA) in various buffer conditions (Phosphate buffer, Tris buffer, Sodium acetate buffer) for

overnight at 4°C. The appropriate condition for assembly was found to be 1:2 molar ratios of F_1 and F_2 and assembly buffer (sodium acetate 0.1M, sodium chloride 0.1M, pH 4.8).

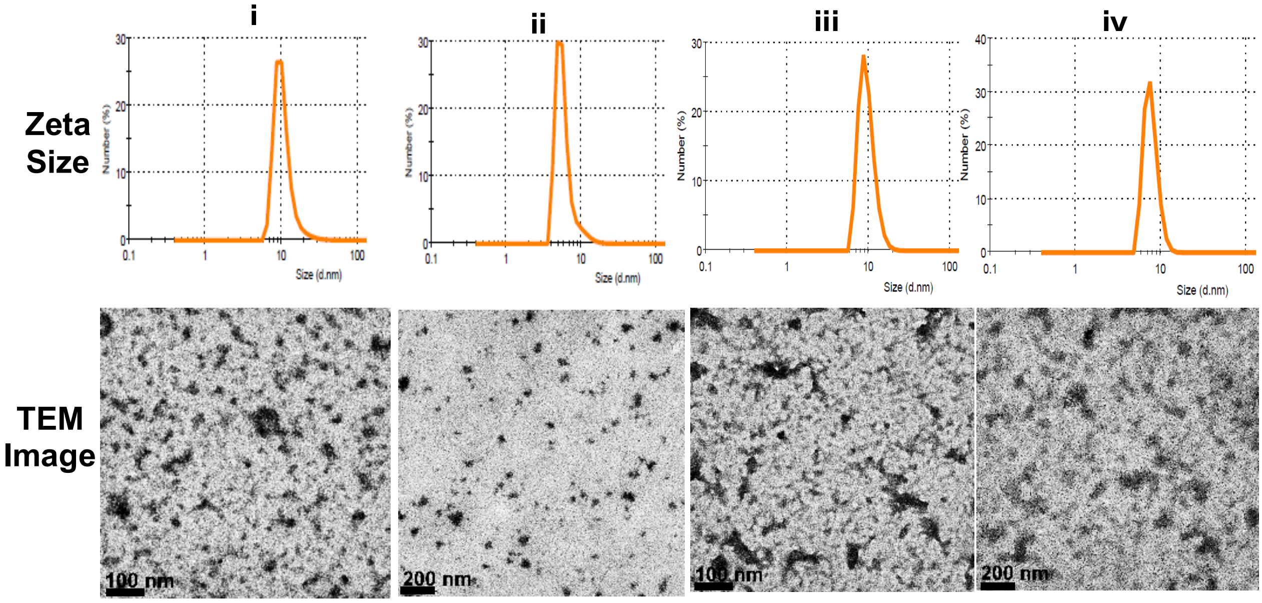

Supplementary Figure 6a: Morphological characterization after purification of (i) F_1 and (ii) F_2 as well as after two months of (iii) F_1 and (iv) F_2 capsid protein. DLS measurement and TEM image of both the capsid protein were taken after the affinity purification (containing 0.25M Imidazole) and after 2 months storage at 4°C in 1x PBS (without *in vitro* assembling conditions. Both the capsid proteins did not show any assembly or aggregation just after the purification.

Zeta size analysis

The average particle size of CCMV capsid was determined using a zeta particle size analyzer based on the DLS principle (Nano ZS 90, Malvern, UK). The measurement of average particle mean diameter of CCMV capsid (1.2 µM) was performed on zeta sizer equipped with a 5 mW helium /neon laser at 4°C in triplicate. The results were expressed as average mean based on the number as variables for particle size distribution (PSD) in dynamic light scattering (DLS).

Transmission electron microscopy (TEM)

The size and morphology of CCMV capsid nanoparticles was observed under TEM. The *in vitro* assembled capsids were diluted to 1.2 µM and about 10 µl of dispersion was placed on carbon-coated copper grid. The samples were stained with 0.1% phosphotungstic acid (PTA) solution for 30 sec. The copper grid was allowed to dry at ambient temperature and observed for TEM (FEI Tecnai G20, Germany). The mean diameter of assembled particles was calculated using Image J software.

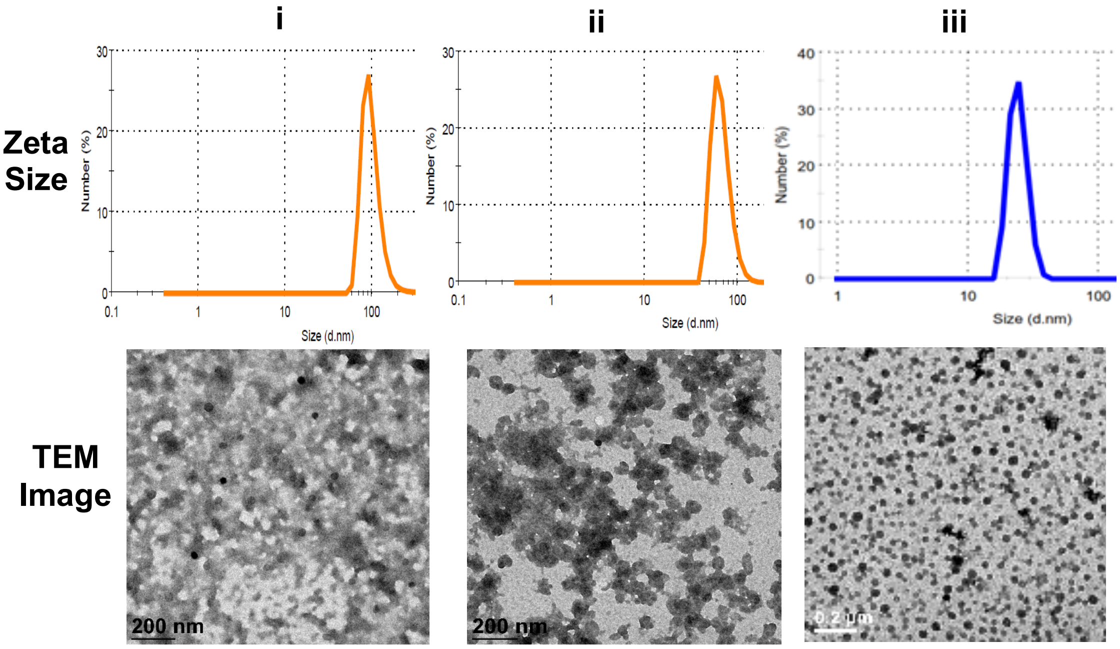

Supplementary Figure 6b: Morphological characterization of (i) F_1, (ii) F_2 and (iii) F_1:F_2 combination *in vitro* assembly condition. DLS analysis and TEM imaging were performed after dialyzing the equimolar concentration of F_1, F_2 and 1:2 ratio of F_1:F_2 capsid protein overnight in assembly buffer (Sodium acetate 0.1M, sodium chloride 0.1M, pH 4.8). Scale bar were mentioned in each figures.

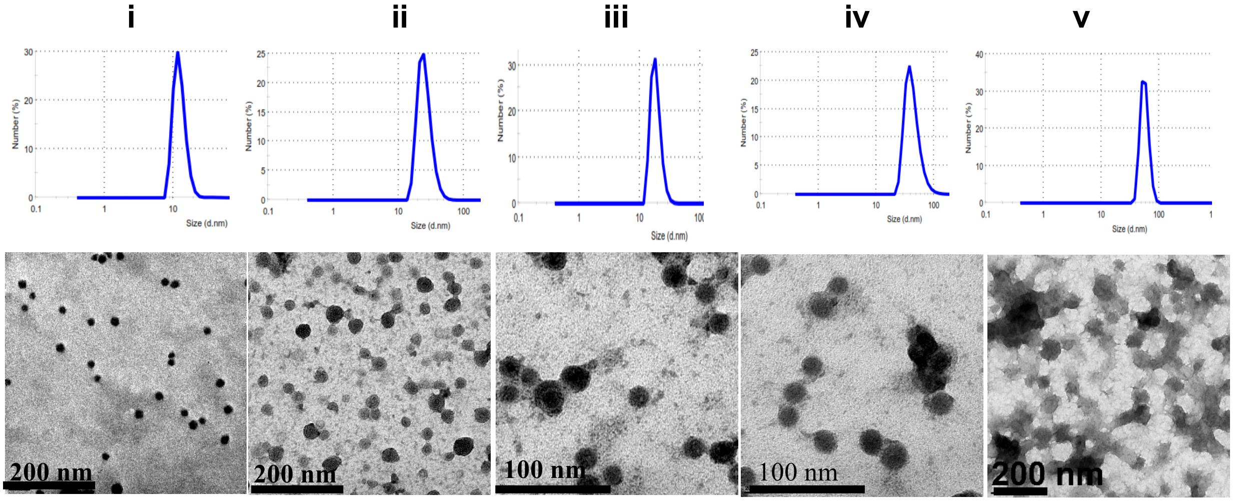

Supplementary Figure 6c: Stability of *in vitro* assembled virus particles at pH range 4.0-8.0. DLS (upper) and TEM (lower) results of *in vitro* assembled VNPs after incubating for 24 hours in dialysis condition at pH (i) 4.0, (ii) 5.0, (iii) 6.0, (iv) 7.0, and (v) 8.0. The particle size at pH 4, 5 and 6 remains same while swelling was observed at pH 7 and 8.

Supplementary Figure 7: Fluorescent dye bioconjugation

Work Curve plot

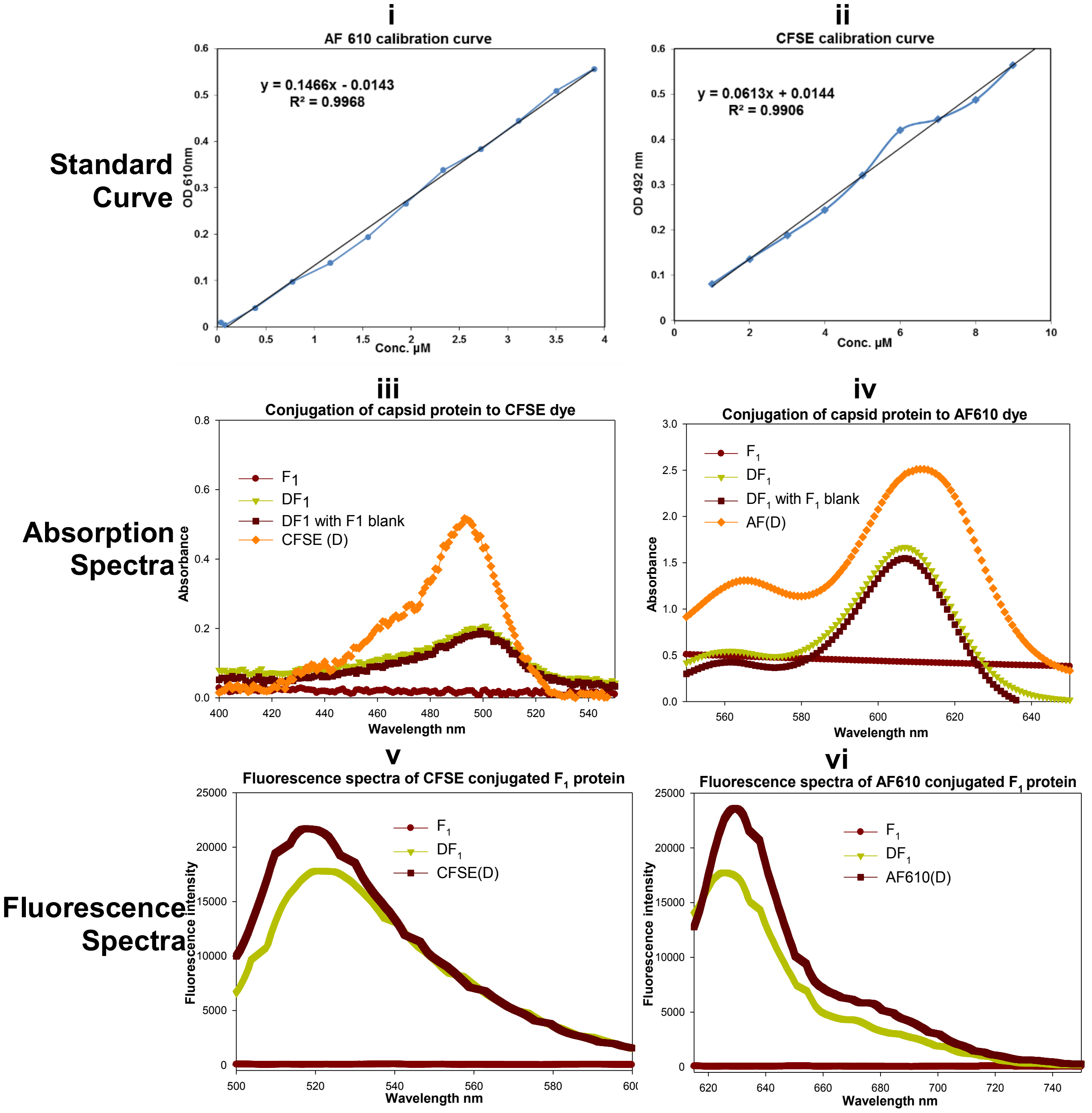

Supplementary Figure 7a: Spectroscopic characterization of (i) CFSE and (ii) AF610 dyes conjugated capsid protein (F_1): Calibration curve using standard concentrations of CFSE (CFSE-D) and AF610 (AF-D) dyes were generated to quantify the dye bioconjugation efficiency. Absorbance spectra of CFSE and AF610 showed characteristic peaks at 492 nm and 610nm respectively. The capsid protein (F_1, non-conjugated) does not showed absorption at either 492nm or 610nm. However, dye conjugated capsid protein (DF_1) showed the characteristic absorption peak of corresponding dyes. These peaks remain while the absorbance was taken using equimolar of non-conjugated capsid proteins. For fluorescence analysis, diluted dye solution, equimolar concentration of native F_1 and completely dialyzed dye conjugated F_1 capsid protein were excited **(**492 nm for CFSE; & 610nm for AF610) and emission spectra were recorded (CFSE: 500 to 650 nm; AF610: 612 to 700 nm). The absence of fluorescence peak in native protein confirms the bioconjugation and it was used to quantify the conjugation efficiency of both the dyes on capsid protein.

The capsid proteins were labeled with carboxyfluorescein succinimidyl ester (CFSE) or Alexa fluor 610 NHS ester (AF610) for fluorescent bio-imaging. The suitable incubation time and bioconjugation efficiency was determined by work curve scan plot. The quantitative estimation of dye bioconjugation was done by work curve absorbance and fluorescence scanning methods with known concentration of dye and F_1 protein. The quantification technique consisted of plotting the absorbance or fluorescence against a series of samples with known concentrations of analyte (standard dye solutions). The standards were dispersed in same suspension buffer corresponding with that of the bioconjugated protein (test samples). The calibration graph was plotted between the concentration of the dye standards on the x-axis and spectrometric measurements in the y-axis (Supplementary Figure 7).

The concentration-data curve of dye standards fit into a straight line using linear regression analysis and explained through the model equation:

$$y = mx + c$$

Where y represented the measurement of spectrometer (absorbance or fluorescence), m denoted for sensitivity and c, a constant described the background. The concentration of dye (x) conjugated on capsid protein (test sample) of unknown concentration could be calculated from the equation.

HPLC Quantification of ligands (dye) Conjugation

Methods

The Dye conjugation was performed using 20:1 molar ratio of dye and F_1 capsid protein through dialysis. The initial equivalent solution (as used in dialysis) of dye and concentration equalized complete dialysate (by speed vac concentration) were analyzed (absorbance at 210nm) by isocratic HPLC (Shimadzu with photodiode detector SPD M20) equipped with C_{18} reverse phase analytical column using ACN:H_2O (30:70) as mobile phase. The concentration equalization means entire dialysate (after complete dialysis) were collected and concentrated to make equal volume used for conjugation reaction. Equal volume (20µl) of both (initial and concentration equalized complete dialysate) were injected through auto sampler in mobile phase equilibrated HPLC for isocratic separation. The standard of each ligand were used to validate the process and protocol of the HPLC.

Results and Discussion

HPLC analysis was performed for the determination of conjugation efficiency for Dye, FA and Doxorubicin on capsid protein. The results were given as suppl. Figure 7b (for dye conjugation), 8d (for FA conjugation), and 9d (for doxorubicin conjugation). The HPLC absorbance chromatogram peaks of initial dye and after bioconjugation were averaged as 1609737 and 1200420 respectively. This confirms that nearly ~26% dye was conjugated on capsid proteins. This is equivalent to 5.2 molar dyes in experimental conditions. Thus, one molecule capsid (used for the experiment) protein has conjugated with 5 molecule of dye with Alexa fluor 610 dye. Based on these and work curve model experiment, it was reported that average 5 molecules were individually bio-conjugated with CP (F_1).

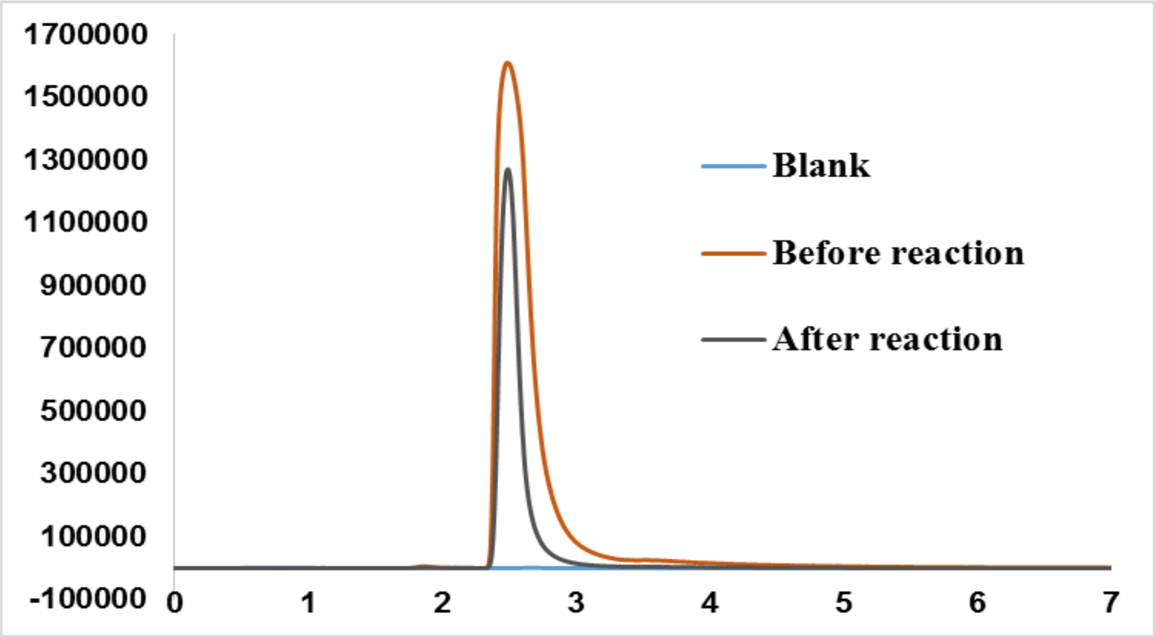

Supplementary Figure 7b: HPLC quantification of Alexa fluor dye bioconjugation. The initial equivalent solution of Alexa Fluor dye and concentration equalized complete dialysate (by speed vac concentration) were analyzed (absorbance at 210nm) by isocratic HPLC (Shimadzu with photodiode detector SPD M20) equipped with C_{18} reverse phase analytical column using ACN:H_2O (30:70) as mobile phase.

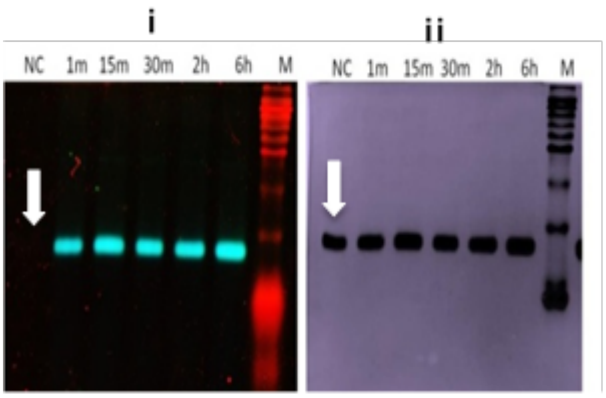

Supplementary Figure 7c: Evaluation of bioconjugation kinetics by SDS PAGE on capsid proteins. Time dependent (1min to 6hrs) labeling of CFSE dye on capsid protein (F_1) loaded on SDS-PAGE Gel. **(i)** The unstained SDS-PAGE gel was visualized using fluorescence gel doc system (FluorChem M, Protein Simple) showed the fluorescent band on dye conjugated F_1 while there is no fluorescence in non-conjugated F_1 protein (indicated by arrow). **(ii)** Same SDS-PAGE gel after coomassie staining showed protein bands on each condition including non-conjugated protein (NC). The dye was incubated with capsid protein for selected time and immediately dialyzed with 1:1000 volume ratio of dialysate. NC: Native non-conjugated capsid protein, M: Molecular weight markers, m: minute incubation time. The incubation time was mentioned in the figures.

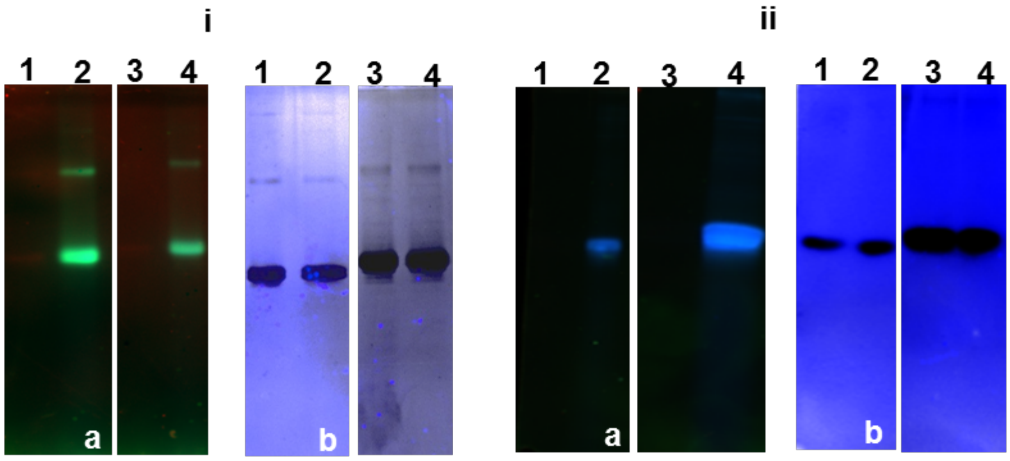

Supplementary Figure 7d: SDS PAGE analysis of (i) CFSE and (ii) AF610 conjugated capsid protein. **(a)** Fluorescence bands of unstained and **(b)** coomassie stained SDS-PAGE gel CFSE after 1 min incubation. Lane 1: Non-conjugated F_1 protein, lane 2: Dye conjugated F_1 protein, lane 3: Non-conjugated F_2 protein, lane 4: Dye conjugated F_2 protein. The fluorescence of conjugated proteins is observed in unstained gel while the non-conjugated protein is only visible in coomassie stained gels at same position.

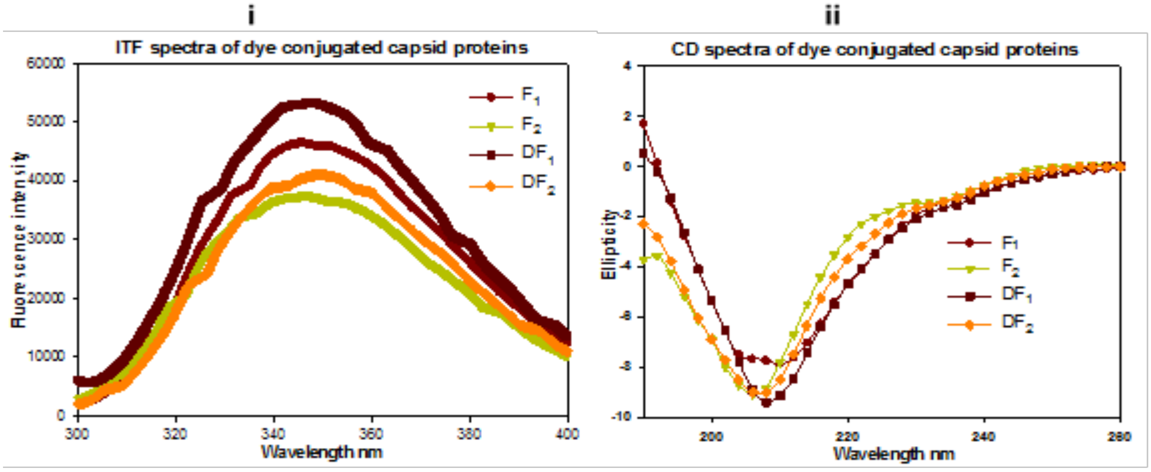

Supplementary Figure 7e: Biophysical characterization of dye bioconjugated capsid proteins. ITF spectra of 0.01mg/ml dye conjugated capsid proteins **(i)** DF_1 and DF_2 were recorded. The desired proteins were excited at 292 nm and emission was taken from 300 to 400 nm. The emission spectra for the capsid proteins were found near 340nm, which confirmed the folded state of proteins. The far UV CD spectra of dye conjugated **(ii)** DF_1 and DF_2 capsid proteins (0.10mg/ml) were taken from 190 to 260nm at ambient temperature. The CD spectra with the negative ellipticity at 208 nm and positive peak below 200nm suggested the folded structure of the proteins. The symbols for different ligands conjugated proteins were given along with each graph.

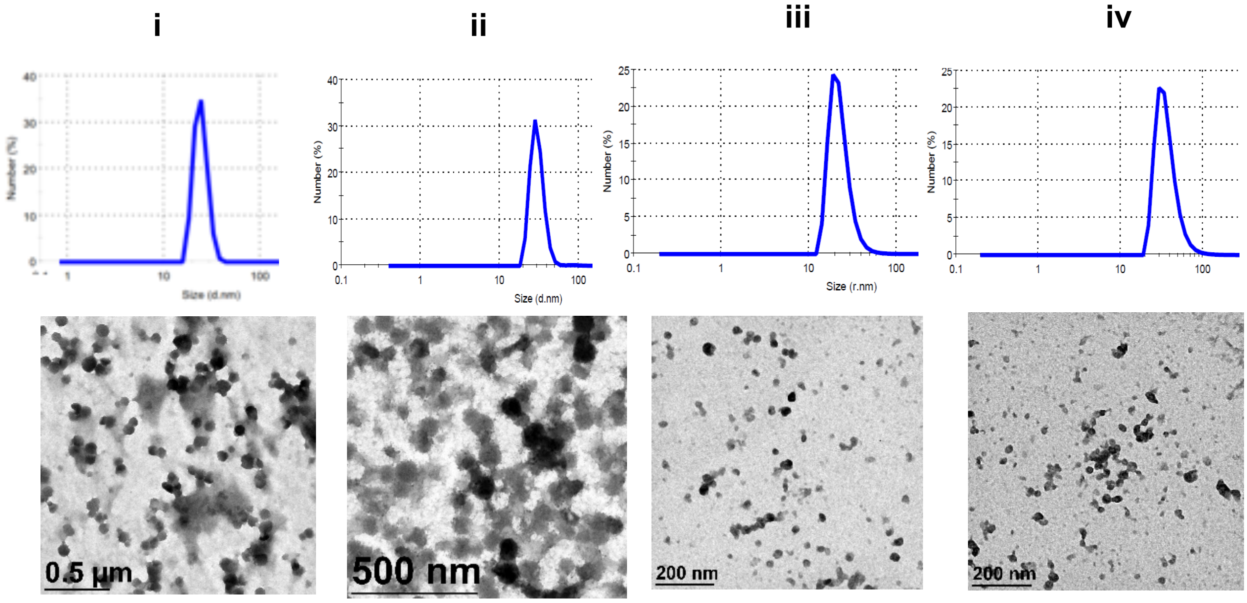

Supplementary Figure 7f: Morphological characterization of *in vitro* assembled CFSE conjugated (i) F_1, (ii) F_2 and AF610 conjugated (iii) F_1 and (iv) F_2 capsid protein. DLS analysis and TEM imaging were performed similar to *in vitro* assembly conditions. Scale bar were mentioned in each figures.

Supplementary Figure 8: Bioconjugation of Folic Acid

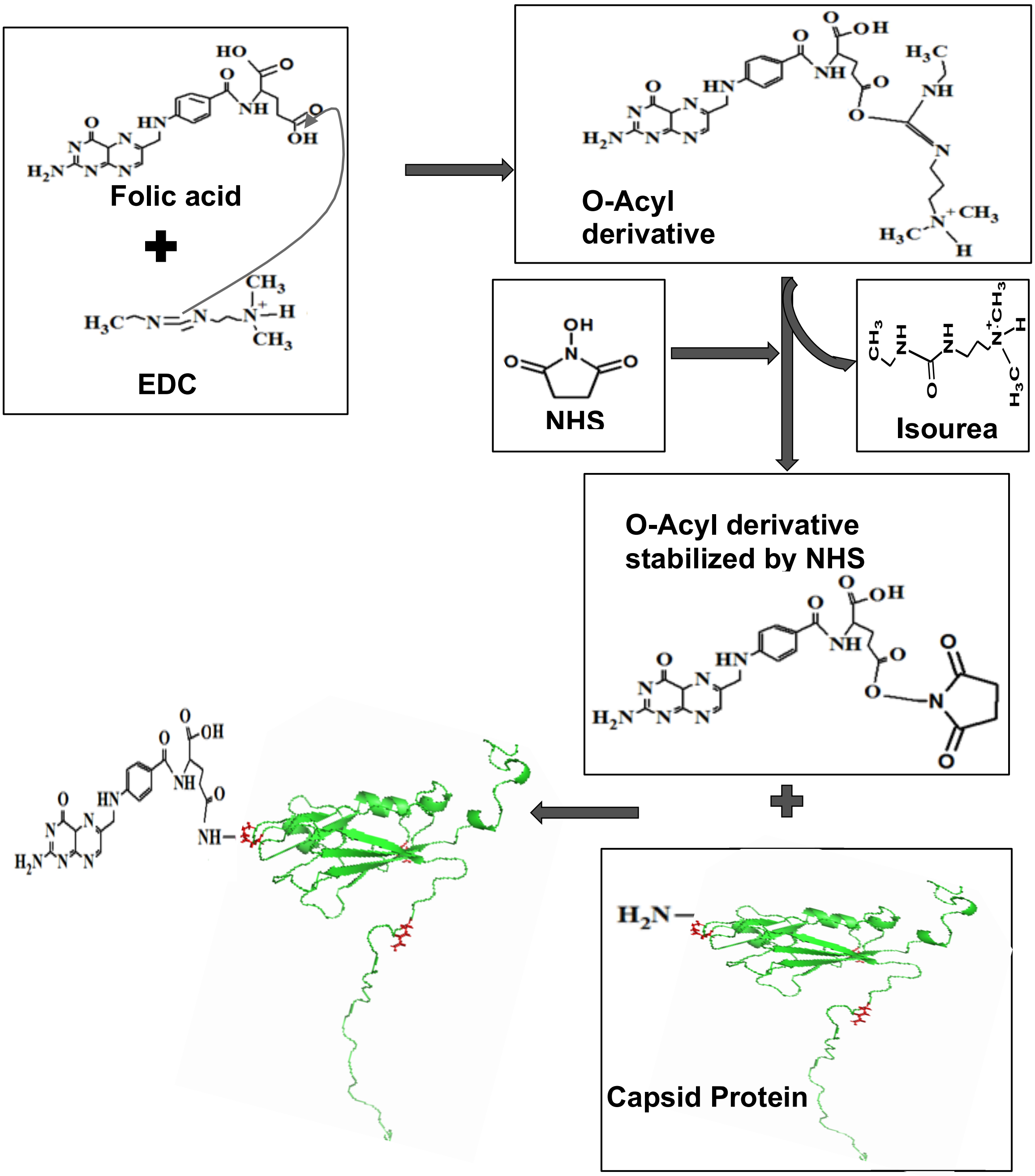

Supplementary Figure 8a: Schematic representation of folic acid bioconjugation to capsid protein by carbodiimide chemistry. Folate molecule was activated by EDC to form O-acyl derivative and further stabilized by NHS. This activated O-acyl derivative of folate was used to react with amine group of lysine residue of capsid protein.

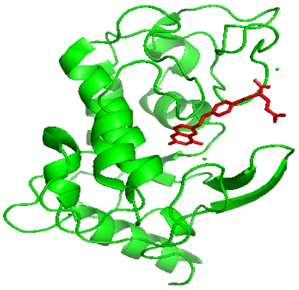

Supplementary Figure 8b: Interaction of folic acid with folate receptor. Figure showing binding of folic acid molecule (red) with folate receptor through pterin ring while the glutamic acid group were available for bioconjugation.

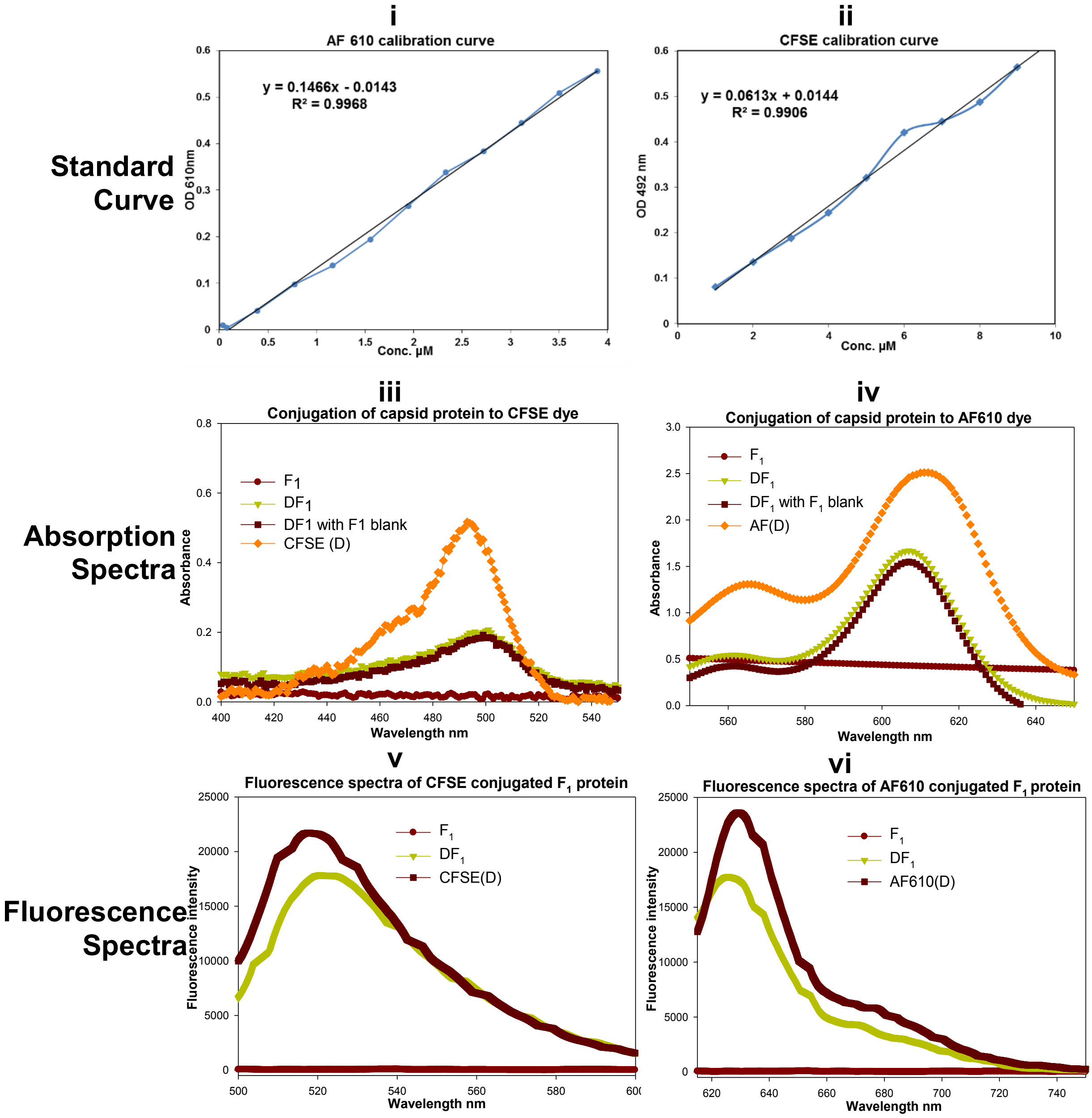

Supplementary Figure 8c: Spectroscopic characterization of folic acid conjugated capsid protein: (i) Calibration curve using standard concentrations of folic acid were generated to quantify the folic acid bioconjugation efficiency. Absorbance spectra of folic acid conjugated (ii) F_1 and (iii) F_2 capsid protein. These spectra were recorded for folic acid (FA), non-conjugated protein (F_1 or F_2) and FA conjugated CCMV capsid proteins ($^{FA+}F_1$ or $^{FA+}F_2$) using PBS as well as equimolar concentration of F_1 or F_2 as blank. The non-conjugated protein does not showed absorption at 363nm. However, folic acid conjugated capsid

proteins ($^{FA+}F_1$ or $^{FA+}F_2$) showed the characteristic absorption peak of folic acid. These peaks remain while the absorbance was taken using equimolar of non-conjugated capsid proteins. The protein was allowed to react with EDC/NHS activated folic acid; $^{FA+}F_1$ and $^{FA+}F_2$ showed peak at 363 nm due to folic acid conjugation same is absent from the non-conjugated proteins.

HPLC Quantification of ligands (FA) Conjugation

Methods.

The FA conjugation was performed using 10:1 molar ratio of dye and F_2 capsid protein through dialysis. The initial equivalent solution (as used in dialysis) of FA and concentration equalized complete dialysate (by speed vac concentration) were analyzed (absorbance at 363nm) by isocratic HPLC (Shimadzu with photodiode detector SPD M20) equipped with C_{18} reverse phase analytical column using ACN:H_2O (30:70) as mobile phase. The concentration equalization means entire dialysate (after complete dialysis) were collected and concentrated to make equal volume used for conjugation reaction. Equal volume (20µl) of both (initial and concentration equalized complete dialysate) were injected through auto sampler in mobile phase equilibrated HPLC for isocratic separation. The standard of each ligand were used to validate the process and protocol of the HPLC.

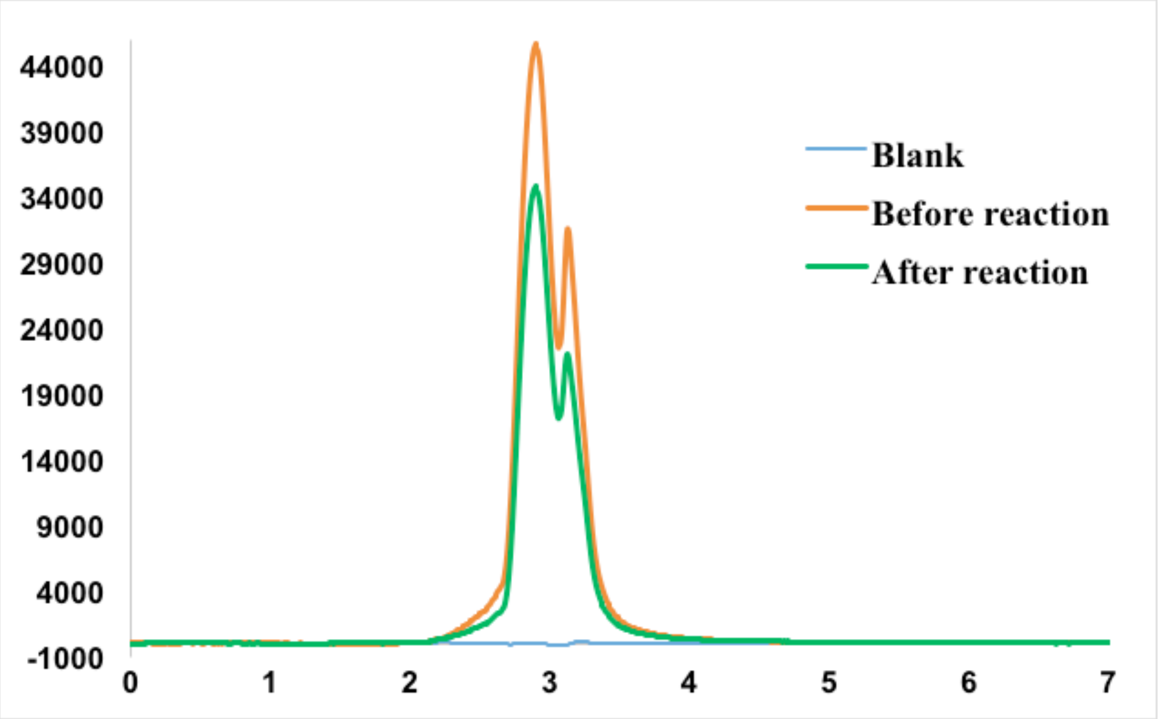

Supplementary Figure 8d: HPLC quantification of folic acid bioconjugation. The initial equivalent solution of folic acid and concentration equalized complete dialysate (by speed vac concentration) were analyzed (absorbance at 363nm) by isocratic HPLC (Shimadzu with photodiode detector SPD M20) equipped with C_{18} reverse phase analytical column using ACN:H_2O (30:70) as mobile phase.

Results and Discussion:

The HPLC absorbance chromatogram peaks of initial FA and after bioconjugation (first peak) were averaged 45544 and 34720 respectively. This confirms that nearly ~23% FA was conjugated on capsid proteins. This is equivalent to 2.3 molar FA in experimental conditions. Thus, one molecule capsid (used for the experiment) protein has conjugated with approximately 2 molecule of FA. Based on these and work curve model experiment, it was reported that average 2 molecules were individually bio-conjugated with CP (F_2).

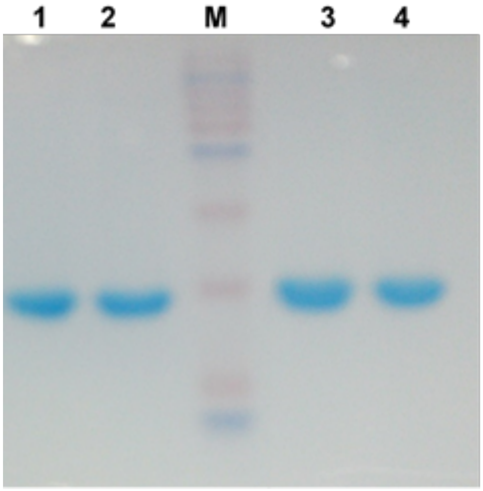

Supplementary Figure 8e: SDS PAGE Gel of FA conjugated proteins ($^{FA+}F_1$ and $^{FA+}F_2$). Lanes: 1 and 3 were F_1 and F_2 capsid proteins; lanes 2 and 4 were FA conjugated $^{FA+}F_1$ and $^{FA+}F_2$ capsid proteins respectively. The conjugated proteins were found intact after FA conjugation.

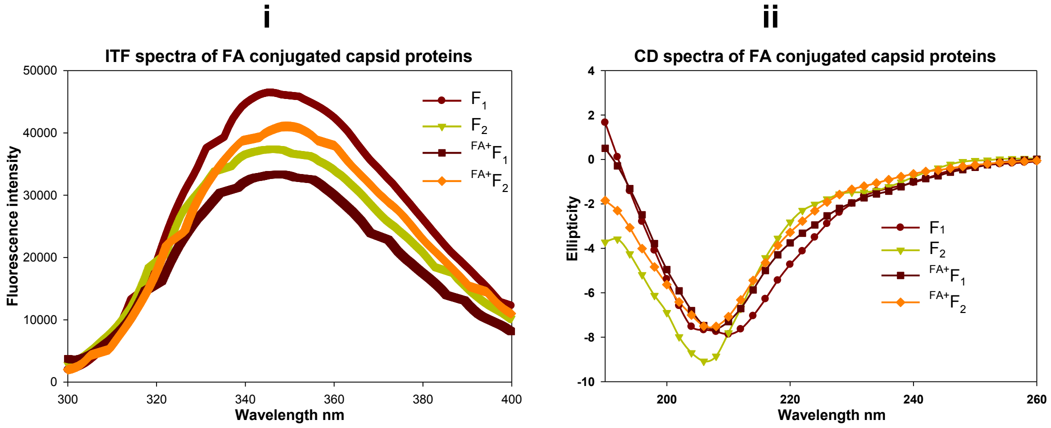

Supplementary Figure 8f: Biophysical characterization by (i) intrinsic tryptophan fluorescence (ITF) and (ii) Far UV CD of folic acid conjugated capsid proteins. For ITF measurements the 0.01mg/ml of F_1, F_2, $^{FA+}F_1$ and $^{FA+}F_2$ (Folic acid conjugated) capsid proteins were excited at 292 nm and emission was recorded from 300 to 400 nm. The protein spectra for the capsid proteins were found near 340nm, which confirmed the folded state of proteins. The Far UV CD spectra of F_1 and F_2 as well as folic acid conjugated ($^{FA+}F_1$ and $^{FA+}F_2$) capsid proteins (0.10mg/ml) were taken from 190 to 260nm at ambient temperature. The CD spectra with the negative ellipticity at 208 nm and positive peak below 200nm suggested the folded structure of the proteins.

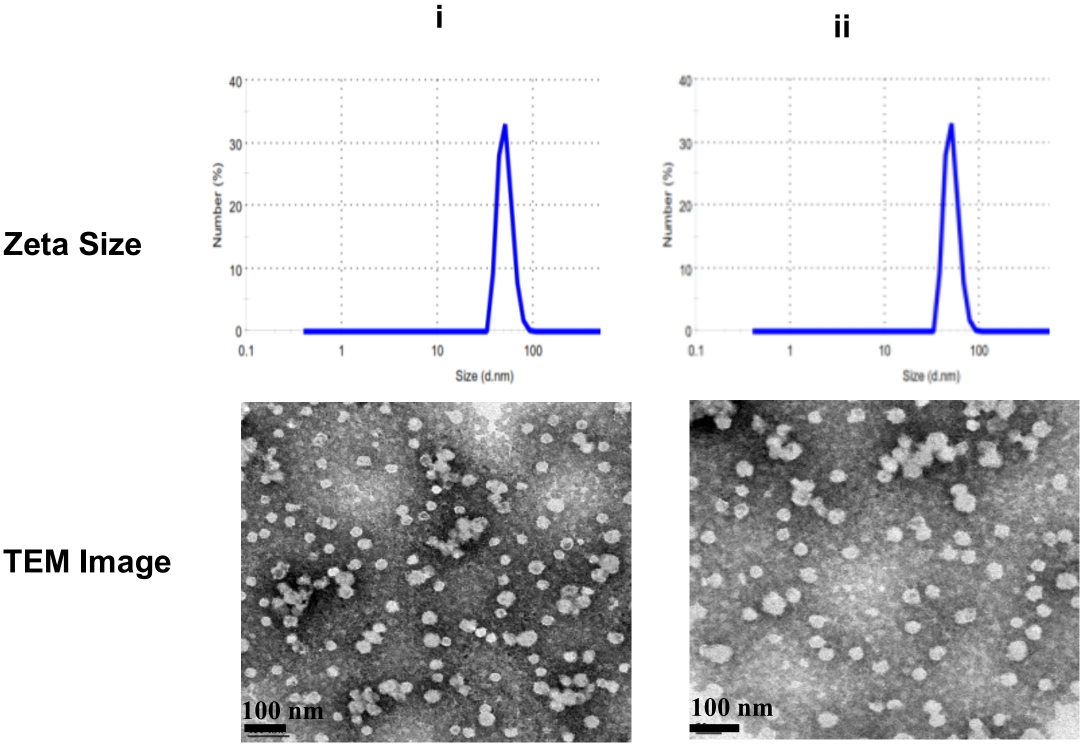

Figure 8g: Morphological characterization of *in vitro* assembled capsid particles of folic acid conjugated proteins. DLS and TEM images of folic acid conjugated CCMV protein F_1 ($^{Fa+}F_1$) **(i)** and F_2 ($^{Fa+}F_2$) **(ii)** respectively. The particles formed were mono- disperse and of similar size as that of particles from non-conjugated protein. DLS analysis and TEM imaging were performed similar to *in vitro* assembly conditions. Scale bar were mentioned in each figures. Scale bar mentioned in the image.

Supplementary Figure 9: Bioconjugation of Doxorubicin

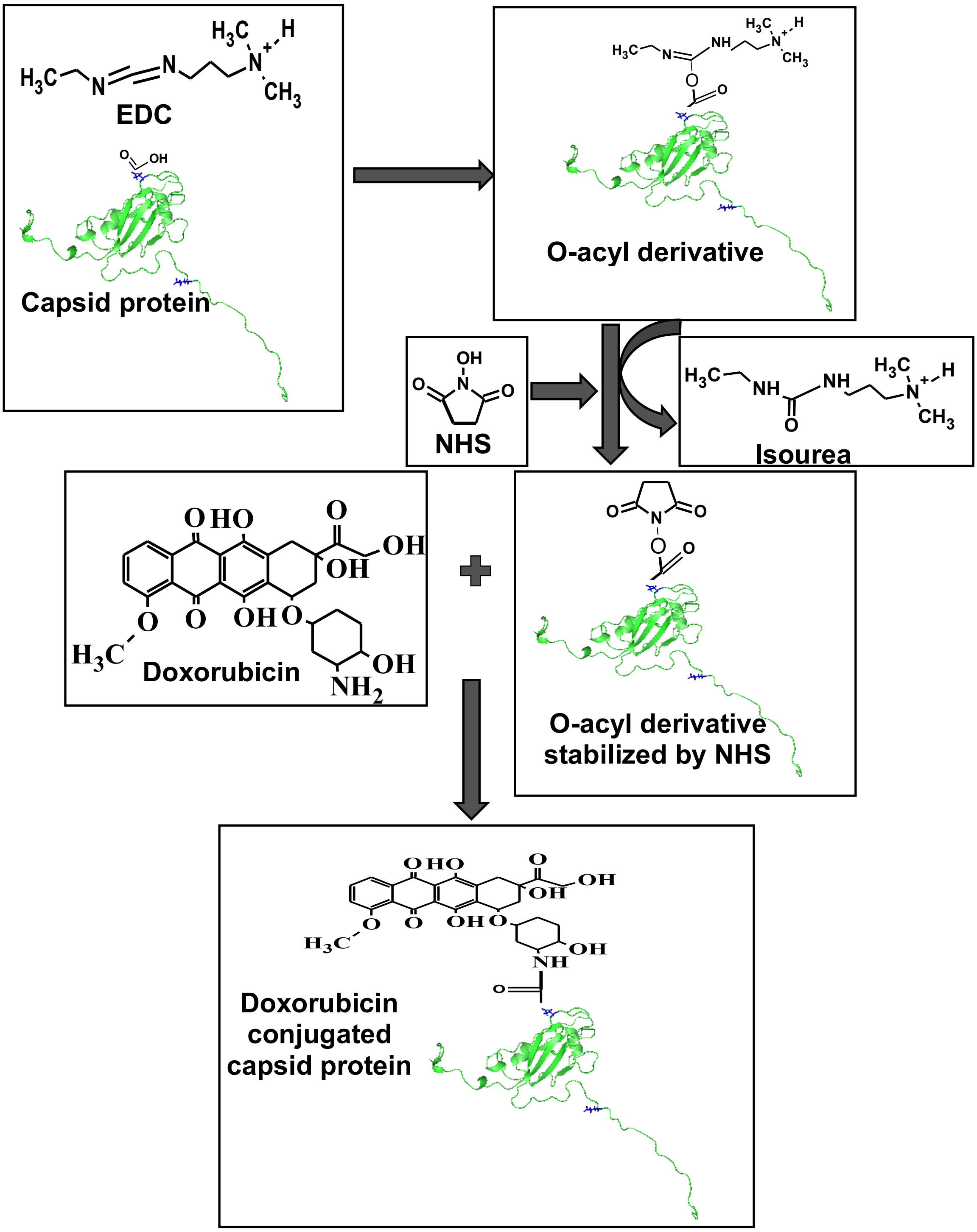

Supplementary Figure 9a: Schematic representation of doxorubicin conjugation on capsid protein by carbodiimide chemistry. The carboxylic group from aspartate and glutamate of capsid protein were activated by EDC to form O-acyl derivative stabilized by NHS. The doxorubicin was bioconjugated with protein in controlled reaction through daunasamine ring.

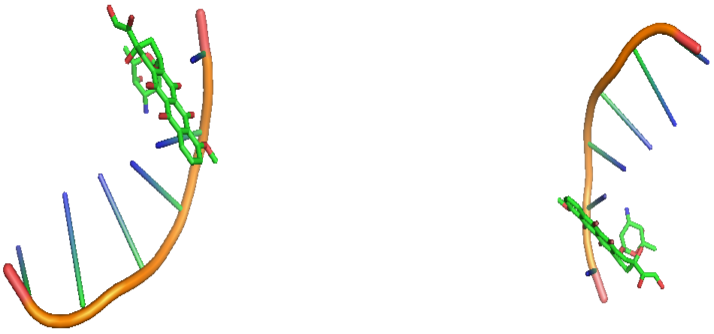

Supplementary Figure 9b: Interaction of doxorubicin with DNA molecule. Doxorubicin (green) was interacted with DNA through anthracycline ring while the amine group of daunosamine sugar can be exploited for bioconjugation to capsid protein.

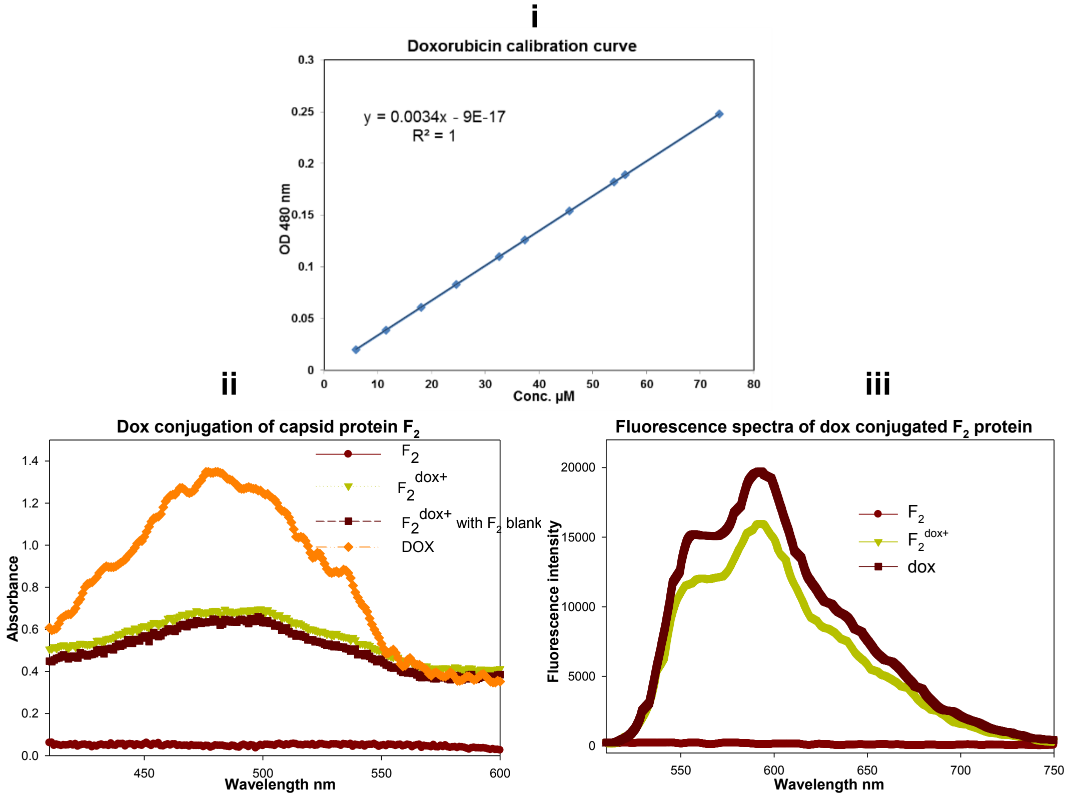

Supplementary Figure 9c: Spectroscopic characterization of doxorubicin conjugated capsid protein: (**i**) Calibration curve for doxorubicin quantification after conjugation by plotting absorbance at 480 nm against standard concentrations. Doxorubicin of known concentration was plotted against absorbance at 480nm. **(ii)** UV-Vis absorbance of doxorubicin (dox), non-conjugated (F_2), conjugated (F_2^{dox+}) and conjugated capsid protein (F_2^{dox+}) against equimolar non-conjugated proteins (F_2) as blank. **(iii)** Fluorescence spectra of doxorubicin conjugated protein (F_2^{dox+}) and non-conjugated (F_2) after excitation at 480nm and emission spectra were recorded from 500 to 750 nm.

HPLC Quantification of ligands (doxorubicin) Conjugation

Methods.

The doxorubicin conjugation was performed using 10:1 molar ratio of doxorubicin and F_2 capsid protein through dialysis. The initial equivalent solution (as used in dialysis) of doxorubicin and concentration equalized complete dialysate (by speed vac concentration) were analyzed (absorbance at 480 nm) by isocratic HPLC (Shimadzu with photodiode detector SPD M20) equipped with C_{18} reverse phase analytical column using ACN:H_2O (30:70) as mobile phase. The concentration equalization means entire dialysate (after complete dialysis) were collected and concentrated to make equal volume used for conjugation reaction. Equal volume (20μl) of both (initial and concentration equalized complete dialysate) were injected through auto sampler in mobile phase equilibrated HPLC for isocratic separation. The standard of each ligand were used to validate the process and protocol of the HPLC.

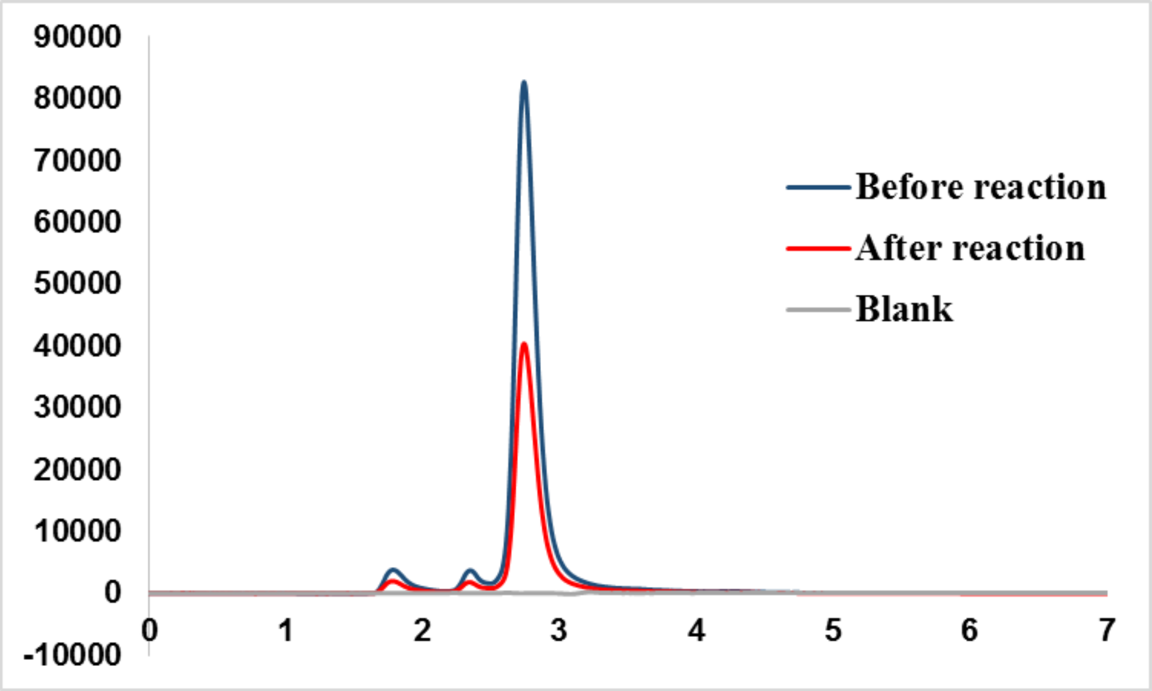

Supplementary Figure 9d: HPLC quantification of doxorubicin bioconjugation. The initial equivalent solution of doxorubicin and concentration equalized complete dialysate (by speed vac concentration) were analyzed (absorbance at 480nm) by isocratic HPLC (Shimadzu with photodiode detector SPD M20) equipped with C_{18} reverse phase analytical column using ACN:H_2O (30:70) as mobile phase.

Results and Discussion:

The HPLC absorbance chromatogram peaks of initial doxorubicin and after bioconjugation were averaged as 80692 and 44369 respectively. This confirms that nearly

~45% doxorubicin was conjugated on capsid proteins. This is equivalent to 4.5 molar doxorubicin in experimental conditions. Thus, one molecule capsid (used for the experiment) protein has conjugated with approximately 4 to 5 molecule of doxorubicin. Based on these and work curve model experiment, it was reported that average 4 doxorubicin molecules were individually bio-conjugated with CP (F_2).

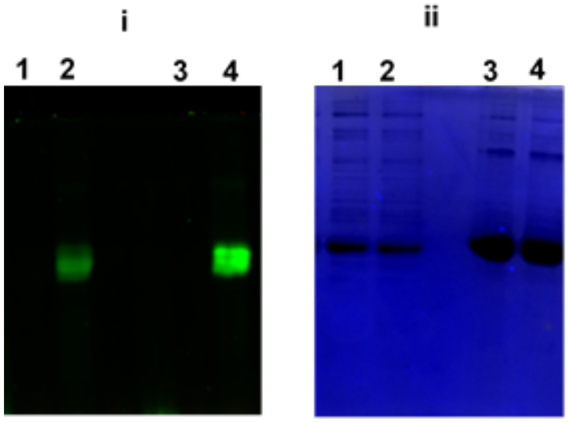

Supplementary Figure 9e: SDS PAGE analysis of doxorubicin conjugated capsid protein. (i) Fluorescence bands of unstained and **(ii)** coomassie stained SDS-PAGE gel. Lane 1: Non-conjugated F_1 protein, lane 2: Doxorubicin conjugated F_1 protein, lane 3: Non-conjugated F_2 protein, lane 4: Doxorubicin conjugated F_2 protein. The fluorescence of conjugated proteins is observed in unstained gel while the non-conjugated protein is only visible in coomassie stained gels at same position.

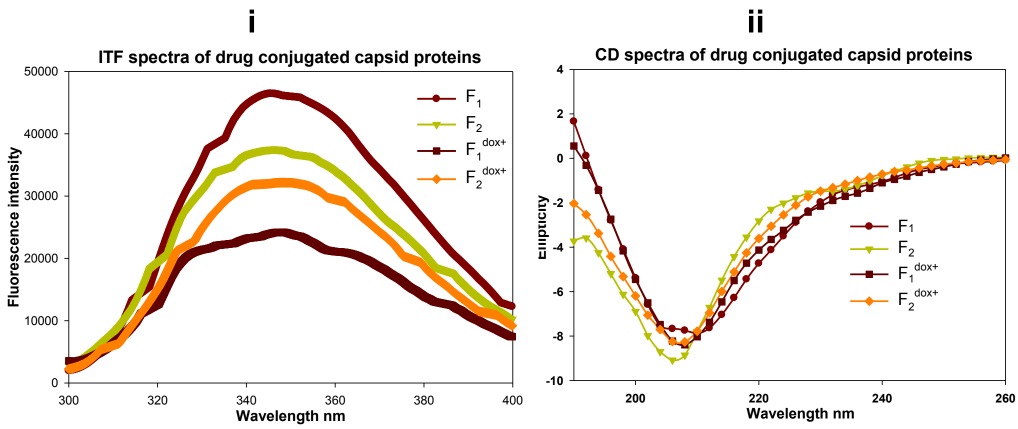

Supplementary Figure 9f: Biophysical characterization by (i) intrinsic tryptophan fluorescence (ITF) and (ii) Far UV CD of doxorubicin conjugated capsid proteins. For ITF measurements the 0.01mg/ml of F_1, F_2, $F_1^{dox+}$and F_2^{dox+} (doxorubicin conjugated) capsid proteins were excited at 292 nm and emission was recorded from 300 to 400 nm. The protein spectra for the capsid proteins were found near 340nm, which confirmed the folded state of proteins. The Far UV CD spectra of F_1 and F_2 as well as folic acid conjugated ($F_1^{dox+}$and F_2^{dox+}) capsid proteins (0.10mg/ml) were taken from 190 to 260nm at ambient temperature. The CD spectra with the negative ellipticity at 208 nm and positive peak below 200nm suggested the folded structure of the proteins.

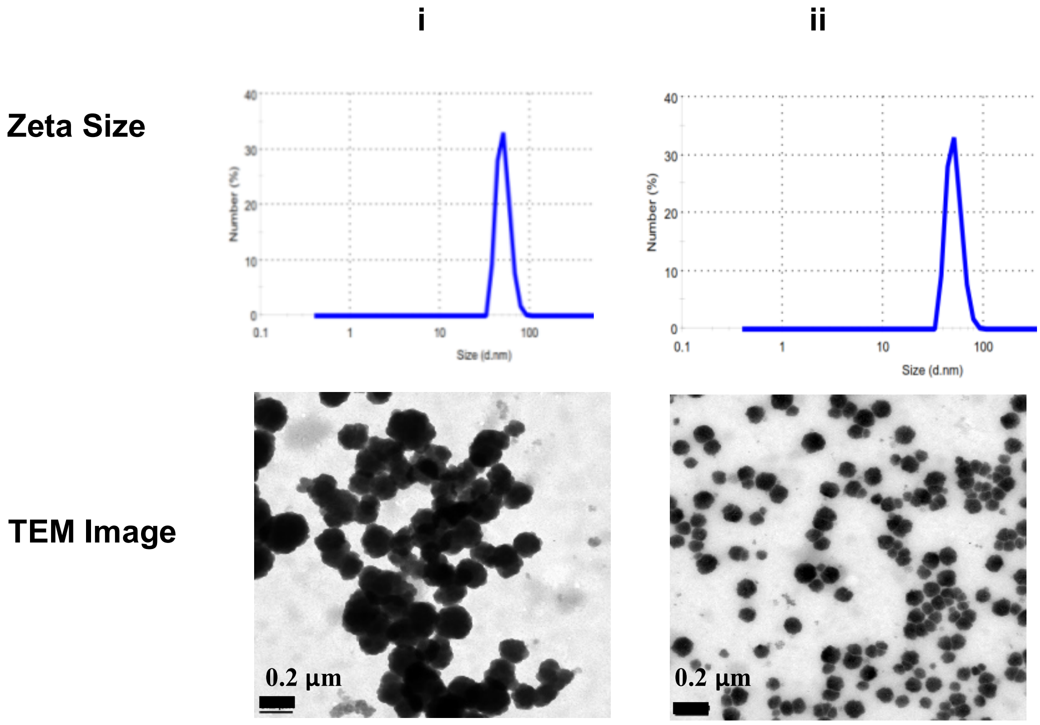

Supplementary Figure 9g: Morphological characterization of *in vitro* assembled capsid particles of doxorubicin conjugated proteins. DLS and TEM images of folic acid conjugated CCMV protein **(i)** (F_1^{dox+}) and **(ii)** (F_2^{dox+}) respectively. The particles were mono- disperse and of similar size as that of particles from non-conjugated protein. DLS analysis and TEM imaging were performed similar to in vitro assembly conditions. Scale bar were mentioned in each figures.

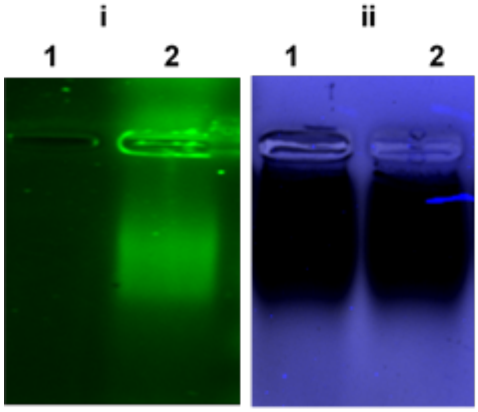

Supplementary Figure 9h: Agarose gel electrophoresis of doxorubicin containing in vitro assembled drug delivery system (DDS). (i) Fluorescence bands of unstained and **(ii)** coomassie stained agarose gel (1%). Lane 1: Non-conjugated DDS and lane 2: Doxorubicin conjugated DDS. The fluorescence of conjugated proteins is observed in unstained gel while the non-conjugated protein is only visible in coomassie stained gels at same position. The *in vitro* assembled DDS with and without doxorubicin were loaded on 1% agarose gel.

Supplementary Figure 10: Functionalization of GNPs with Doxorubicin

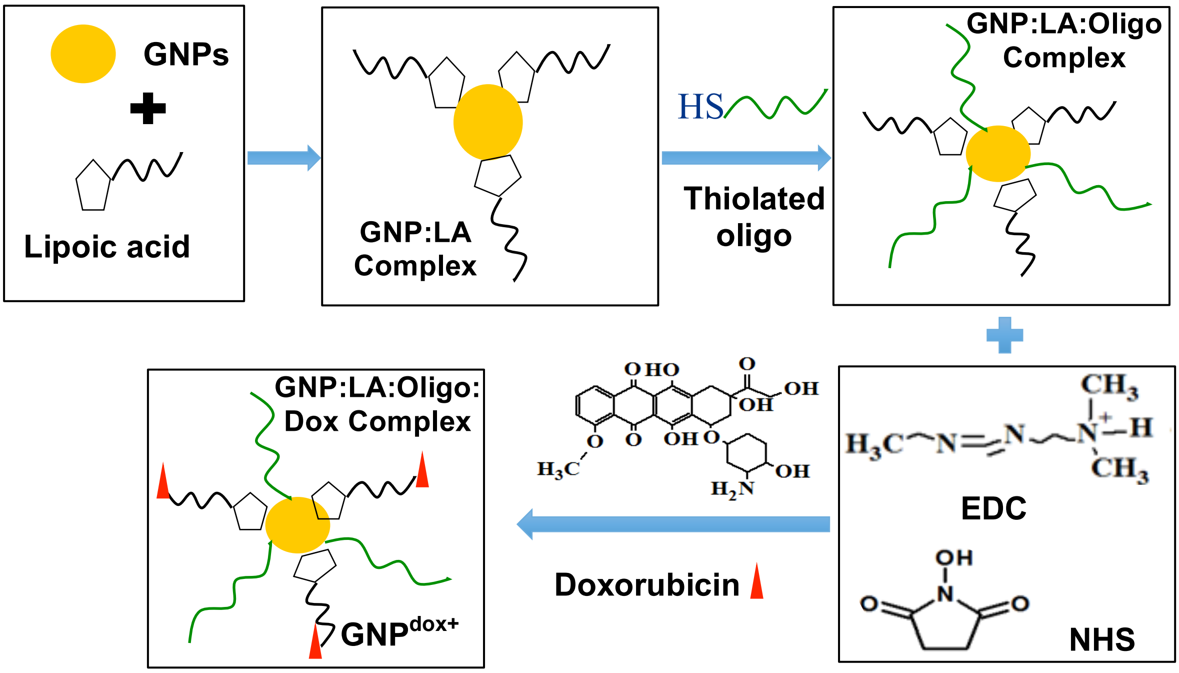

Supplementary Figure 10a: Schematic representation of synthesis of GNP-doxorubicin complex. (i) The lipoic acid (LA) and thiolated oligos were used to functionalise the GNPs to form GNP:LA:SH-Oligo complex. The acidic groups of LA of this complex were activated by EDC NHS chemistry and doxorubicin was added to generate GNP:Dox:LA:SH-Oligo complex denoted as GNP^{dox+}. Thiol group on LA and SH-oligo were exploited for the bioconjugation on GNPs. The oligos has mimicking effect of nucleic acid, which played important role for the encapsidation reaction.

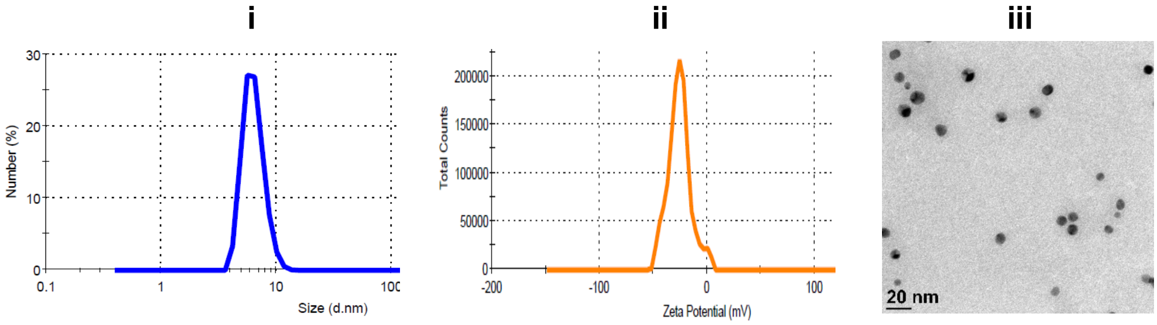

Supplementary Figure 10b: Morphological Characterization of synthesized GNP. (i) Size distribution **(ii)** Zeta potential and **(iii)** TEM image of synthesized GNPs. Mono-disperse GNPs of size 10-12nm were synthesized by sodium borohydride reduction of $HAuCl_4$. Scale bar is mentioned in figure.

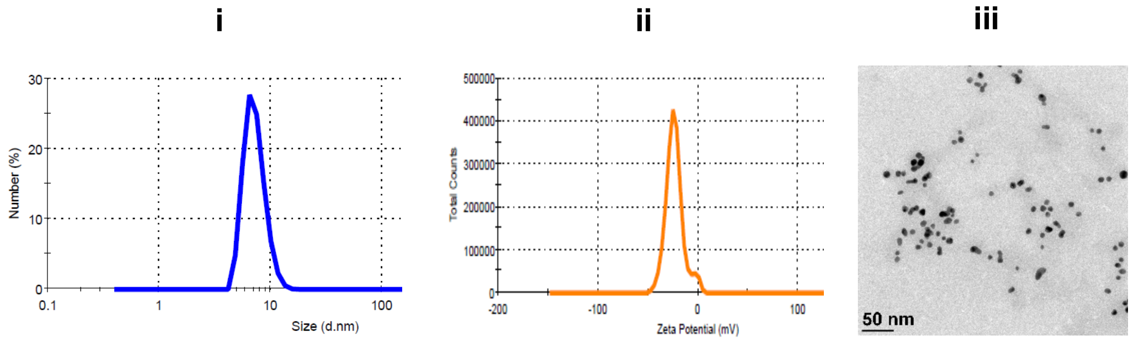

Supplementary Figure 10c: Morphological characterization of doxorubicin conjugated GNP (GNP^{dox+}): (i) Size distribution **(ii)** Zeta potential and **(iii)** TEM image of synthesized GNPs. Scale bar is mentioned in figure.

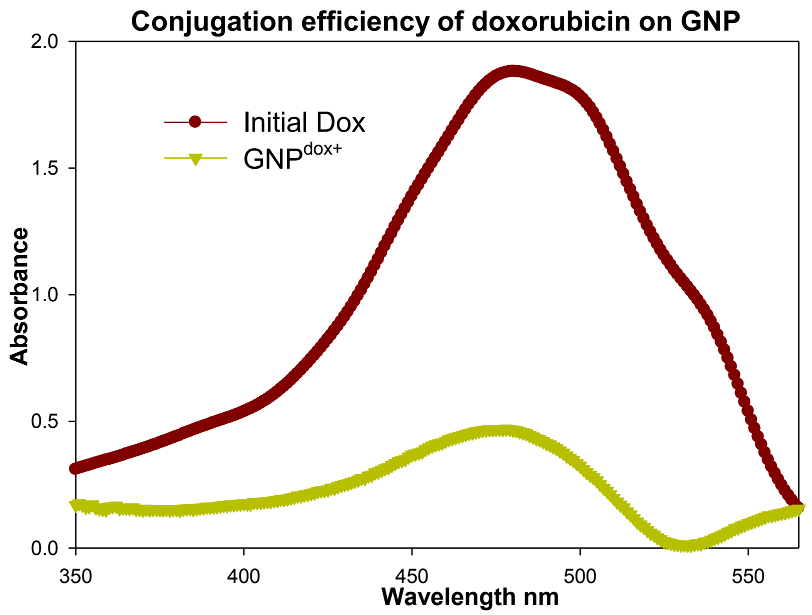

Supplementary Figure 10d: Determination of encapsulation efficiency on GNPs nanoparticles. The complete solution after bioconjugation was extensively dialyzed. The absorption spectrum was recorded for initial doxorubicin solution (Initial Dox) used for conjugation on GNPs. The second spectrum (GNP^{dox+}) was recorded for taking dialyzed GNP^{dox+} complex using equimolar concentration of GNPs as blank. The conjugation efficiency was calculated using the formula given in method section.

Supplementary Figure 11: Stability of synthesized chimeric delivery vehicles

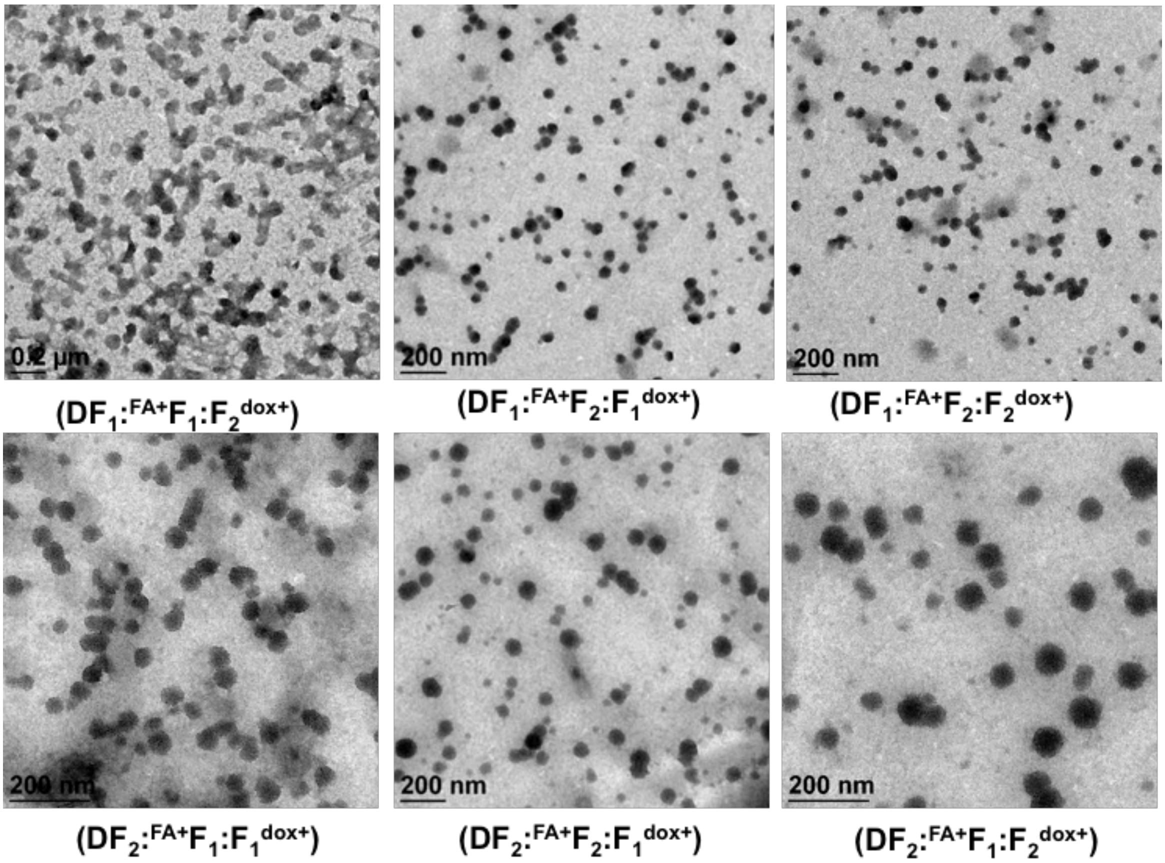

Supplementary Figure 11a: Development of chimeric drug delivery vehicle without GNPs encapsidation. Both capsid proteins (F_1 and F_2) bioconjugated with dye (DF_1 or DF_2), folic acid (**$^{FA+}F_1$ or $^{FA+}F_2$**) and doxorubicin (**F_1^{dox+} or F_2^{dox+}**) were in vitro assembled in a controlled approach using all possible combination by maintaining F_1:F_2::1:2 molar ratio. This helps in identifying the best combination for development of such vehicles. The best combination was recoded with the condition **DF_1 :$^{FA+}F_2$:F_2^{dox+}.** The ligands conjugated with the capsid proteins were given along with each figure, which was used for *in vitro* assembly.

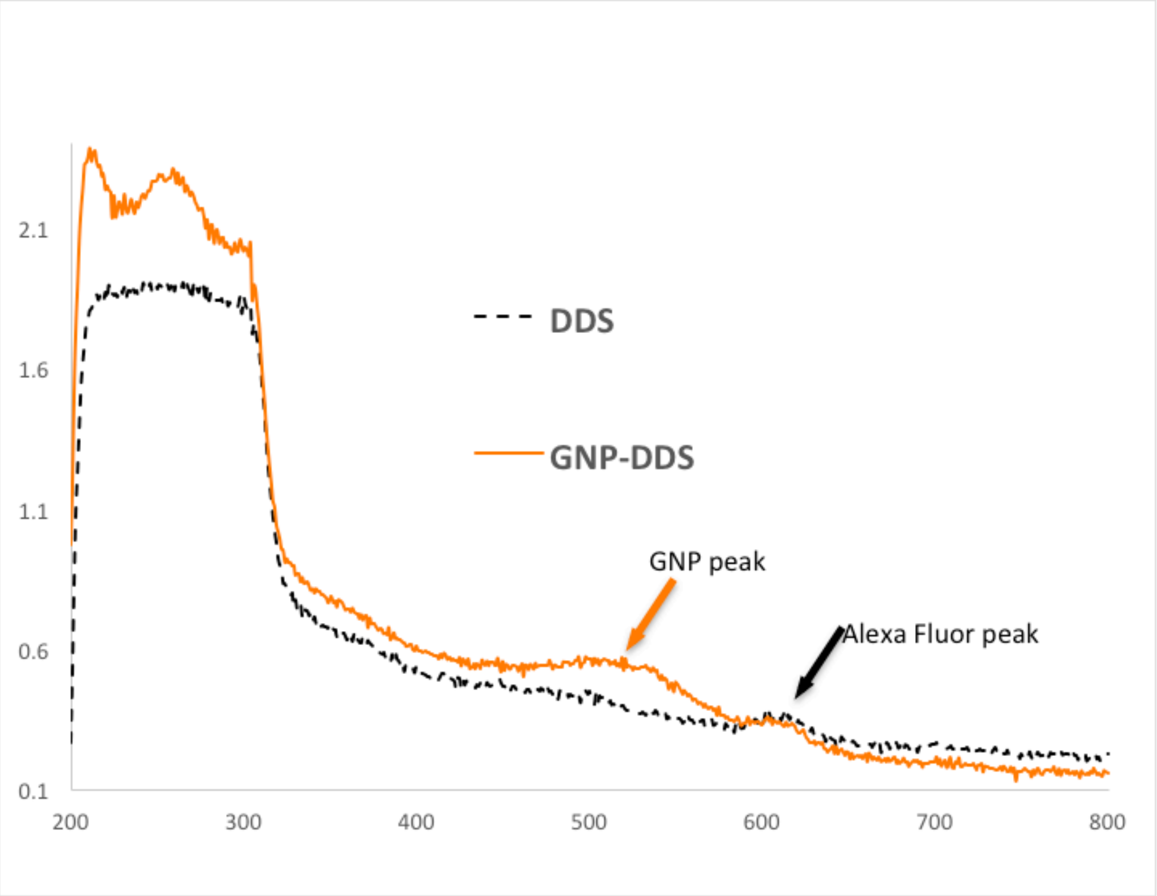

Supplementary Figure 11b: Storage stability of DDS (without GNPs encapsidation) and with GNPs encapsidated (GNP^{dox+}:$^{FA+}DDS^{dox+}$) after 4 months incubation at 4°C. Similar molar capsid concentration of both the capsid (DDS and GNP^{dox+}:$^{FA+}DDS^{dox+}$) were incubated and absorbance were taken after four month. The relatively higher reduction in concentration of DDS in comparison to GNP^{dox+}:$^{FA+}DDS^{dox+}$ indicates that GNPs encapsidation increase the stability of GNP^{dox+}:$^{FA+}DDS^{dox+}$.

Supplementary Table 2: Complete detail of the concentration of native and ligands bioconjugated capsid proteins to synthesize different variants of chimeric delivery vehicles

S.No	DDS Name	Final concentration of native and ligands bioconjugated capsid proteins (µM)							
		F_1	DF_1	$^{FA+}F_1$	F_1^{dox+}	F_2	DF_2	$^{FA+}F_2$	F_2^{dox+}
1	DF_1:$^{FA+}F_1$:F_2^{dox+}	0.8	1.7	1.7	-	3.36	-	-	5.04
2	DF_1:$^{FA+}F_2$:F_1^{dox+}	0.8	1.7	-	1.7	3.36	-	5.04	-
3	DF_1:$^{FA+}F_2$:F_2^{dox+}	2.5	1.7	-	-	1.68	-	3.36	3.36
4	DF_2:$^{FA+}F_1$:F_1^{dox+}	0.8	-	1.7	1.7	3.36	5.04	-	-
5	DF_2:$^{FA+}F_2$:F_1^{dox+}	2.5	-	-	1.7	1.68	3.36	3.36	-
6	DF_2:$^{FA+}F_1$:F_2^{dox+}	2.5	-	1.7	-	1.68	3.36	-	3.36

Supplementary Figure 12: Cellular uptake of DDS under various conditions

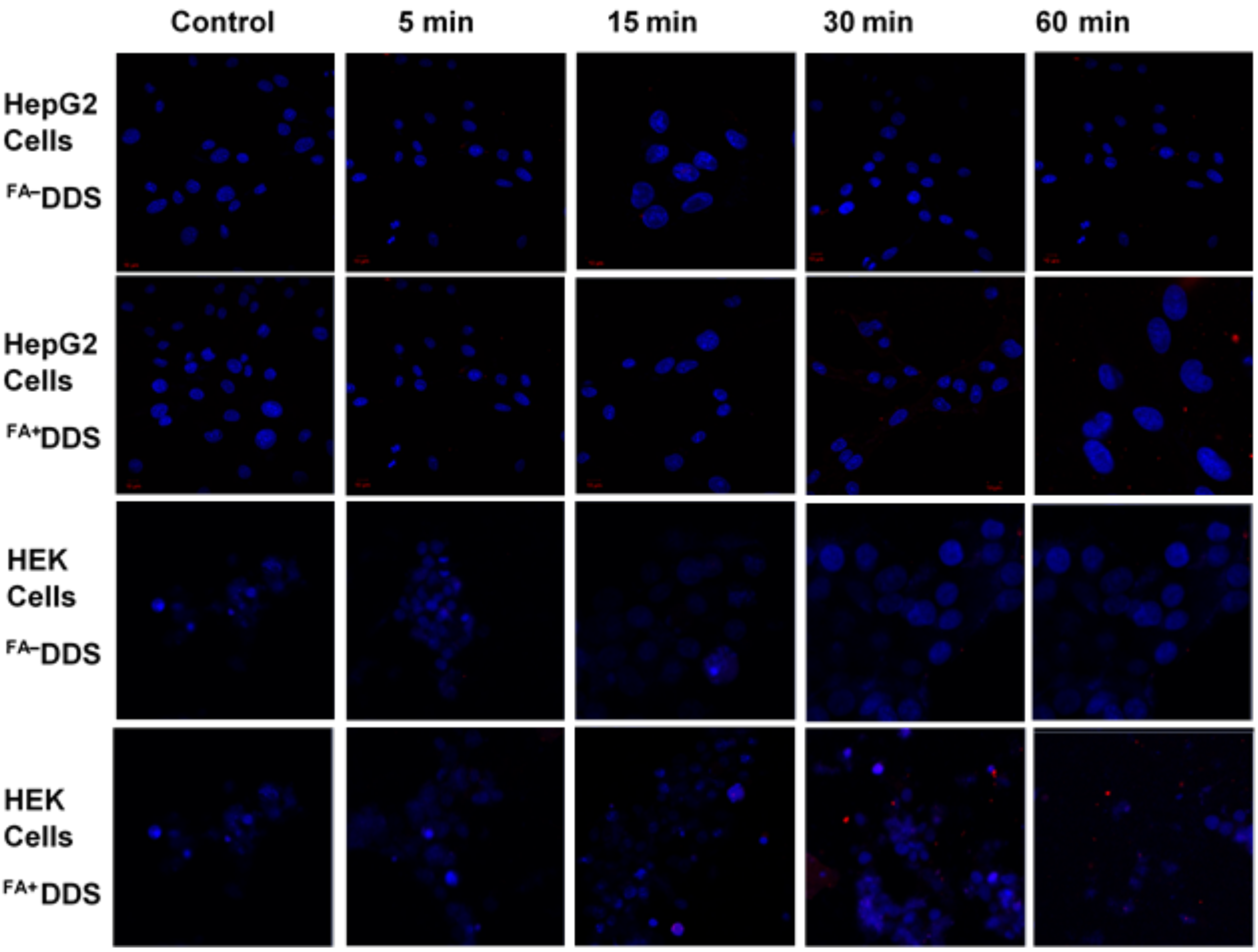

Supplementary Figure 12a: *In vitro* cellular uptake of $^{FA+}$DDS and $^{FA-}$DDS by FR$^-$ HepG2 cells and HEK cells. The DDS were conjugated with fluorescent dye AF610. The cells were incubated for selected period of time (5, 15, 30, and 60min) with folic acid conjugated ($^{FA+}$DDS) and folic acid non-conjugated ($^{FA-}$DDS) drug delivery systems. There was negligible internalization (no fluorescence) due to absence of folate receptors in these cell lines.

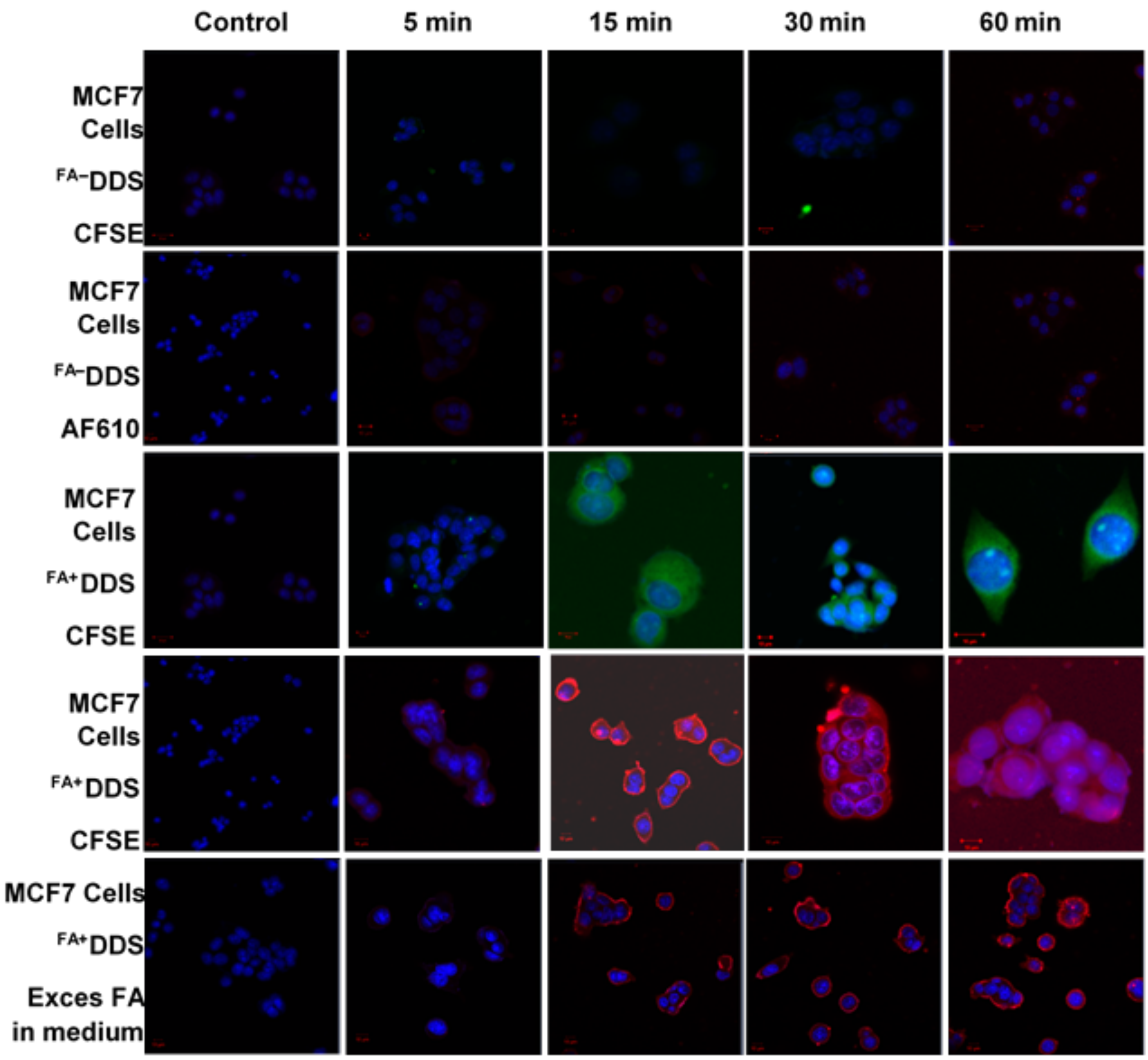

Supplementary Figure 12b: *In vitro* cellular uptake of $^{FA+}$DDS and $^{FA-}$DDS by FR^{+} MCF7 cells by confocal microscope imaging. The DDS were conjugated with fluorescent dye CFSE and AF610. The cells were incubated for selected period of time (5, 15, 30, and 60min) with folic acid conjugated ($^{FA+}$DDS) and folic acid non-conjugated ($^{FA-}$DDS) drug delivery systems. There was internalization (fluorescence) in only $^{FA+}$DDS due to presence of folic acid on DDS and folate receptors on MCF7 cell lines. Confocal micrograph of excess of folic acid in the medium to competitively block the folate receptor of MCF7 cells. The fluorescence was recorded only on the surface of cells containing excess of FA in the medium.

References:

1 Haas, J., Park, E.-C. & Seed, B. Codon usage limitation in the expression of HIV-1 envelope glycoprotein. *Current Biology* **6**, 315-324, doi:http://dx.doi.org/10.1016/S0960-9822(02)00482-7 (1996).

2 Allison, R. F., Janda, M. & Ahlquist, P. Sequence of cowpea chlorotic mottle virus RNAs 2 and 3 and evidence of a recombination event during bromovirus evolution. *Virology* **172**, 321-330 (1989).

3 Speir, J. A., Munshi, S., Wang, G., Baker, T. S. & Johnson, J. E. Structures of the native and swollen forms of cowpea chlorotic mottle virus determined by X-ray crystallography and cryo-electron microscopy. *Structure* **3**, 63-78 (1995).

4 Zhao, X., Fox, J. M., Olson, N. H., Baker, T. S. & Young, M. J. In vitro assembly of cowpea chlorotic mottle virus from coat protein expressed in Escherichia coli and in vitro-transcribed viral cDNA. *Virology* **207**, 486-494 (1995).

5 Vriend, G., Hemminga, M., Verduin, B., De Wit, J. & Schaafsma, T. Segmental mobility involved in protein—RNA interaction in cowpea chlorotic mottle virus. *FEBS letters* **134**, 167-171 (1981).

6 Adolph, K. W. & Butler, P. J. Assembly of a spherical plant virus. *Philos Trans R Soc Lond B Biol Sci* **276**, 113-122 (1976).

7 Adolph, D. W. & Butler, P. J. Studies on the assembly of a spherical plant virus. III. Reassembly of infectious virus under mold conditions. *J Mol Biol* **109**, 345-357 (1977).

8 Roy, A., Kucukural, A. & Zhang, Y. I-TASSER: a unified platform for automated protein structure and function prediction. *Nat. Protocols* **5**, 725-738 (2010).

9 Lee, H., Park, H., Ko, J. & Seok, C. GalaxyGemini: a web server for protein homo-oligomer structure prediction based on similarity. *Bioinformatics* **29**, 1078-1080, doi:10.1093/bioinformatics/btt079 (2013).

10 Zhang, H. G. *et al.* Addition of six-His-tagged peptide to the C terminus of adeno-associated virus VP3 does not affect viral tropism or production. *Journal of virology* **76**, 12023-12031 (2002).

11 Studier, F. W., Rosenberg, A. H., Dunn, J. J. & Dubendorff, J. W. [6] Use of T7 RNA polymerase to direct expression of cloned genes. *Methods in enzymology* **185**, 60-89 (1990).

12 Oliveira, M., Coutinho, J., Krieger, J., Raw, I. & Ho, P. Site-directed mutagenesis of bovine FGF-2 cDNA allows the production of the human-form of FGF-2 in Escherichia coli. *Biotechnology letters* **23**, 1151-1157 (2001).

13 Magnan, C. N., Randall, A. & Baldi, P. SOLpro: accurate sequence-based prediction of protein solubility. *Bioinformatics* **25**, 2200-2207 (2009).

14 Fling, S. P. & Gregerson, D. S. Peptide and protein molecular weight determination by electrophoresis using a high-molarity tris buffer system without urea. *Analytical biochemistry* **155**, 83-88 (1986).
